# Human 3D liver spheroids support productive infection of a novel tick-borne phenuivirus

**DOI:** 10.1016/j.onehlt.2026.101321

**Published:** 2026-01-10

**Authors:** Wenbo Xu, Liyan Sui, Nan Liu, Lesley Bell-Sakyi, Yicheng Zhao, Yuanzhi Wang, Yinghua Zhao, Changfeng Zhu, Quan Liu

**Affiliations:** a Department of Infectious Diseases and Center for Infectious Diseases and Pathogen Biology, Key Laboratory of Organ Regeneration and Transplantation of the Ministry of Education, State Key Laboratory for Diagnosis and Treatment of Severe Zoonotic Infectious Diseases, Key Laboratory for Zoonosis Research of the Ministry of Education, The First Hospital of Jilin University, Changchun, China; b Chinese Medicine Guangdong Laboratory /State Key Laboratory of Traditional Chinese Medicine Syndrome, The Second Clinical College, Guangzhou University of Chinese Medicine (Guangdong Provincial Hospital of Chinese Medicine), Guangzhou, China; c Department of Infection Biology and Microbiomes, Institute of Infection, Veterinary and Ecological Sciences, University of Liverpool, Liverpool, Science Park IC2, 146 Brownlow Hill, Liverpool L3 5RF, United Kingdom; d Department of Pathogenic Biology, School of Medicine, Shihezi University, Shihezi, Xinjiang Uygur Autonomous Region, China; e Department of Gastroenterology and Hepatology, Zhongshan Hospital, Fudan University, Shanghai, China

**Keywords:** Human 3D liver spheroids, Phenuivirus, Mukawa virus, Infection model, Public health

## Abstract

The identification of novel tick-borne viruses, such as Mukawa virus (MKWV), underscores a growing need to assess their potential public health risks. In this study, we isolated the MKWV strain HLJ1 from *Ixodes persulcatus* ticks. While this initial isolate demonstrated limited replication in mammalian cell lines and mice, it productively infected human primary cell-derived 3D spheroids. Serial passaging in this model significantly enhanced viral titers, suggesting adaptive evolution. The resulting adapted strain exhibited increased virulence, causing pronounced cytopathic effects in Vero cells, infecting diverse mammalian cell types, and leading to 100% mortality in suckling mice, with associated liver inflammation and damage. These pathogenic outcomes were recapitulated in the 3D human liver spheroids, which showed impaired cellular synthetic functions, cell death, and heightened inflammatory responses following infection. Epidemiological screening of 145 serum samples from tick-bitten patients in Northeastern China revealed low but detectable exposure, with 1.4% positive for MKWV RNA, 4.8% for IgG antibodies, and 3.4% for neutralizing antibodies. Collectively, our findings integrate a novel human-relevant 3D culture system with field surveillance to highlight the potential risks of MKWV to human health and provide a model framework for evaluating emerging tick-borne viruses.

## 1 Introduction

Tick-borne viral diseases have emerged a critical global public health threat in recent decades [1]. In China alone, at least ten tick-borne viruses have been identified, including severe fever with thrombocytopenia syndrome virus (SFTSV), Jingmen tick virus, Alongshan virus (ALSV), Tacheng tick virus 1 and 2, Songling virus, Beiji nairovirus (BJNV), Yezo virus, Karshi virus, and Guertu virus (GTV) [[2], [3], [4], [5], [6], [7], [8], [9], [10], [11]]. The expanding diversity of these pathogens underscores a growing and serious risk to public health. Mukawa virus (MKWV), a novel tick-borne phenuivirus phylogenetically related to mosquito- and sandfly-borne phleboviruses, exemplifies this threat [12]. Initially detected in *Ixodes persulcatus* ticks in Hokkaido, Japan [13], MKWV has since been reported in Heilongjiang and Jilin Provinces in northeastern China [14,15]. Although no confirmed human infections have been documented, in vitro studies show MKWV can replicate in human hepatoma and tick cells, and induce cytopathic effects (CPE) in Vero cells [13]. Furthermore, the virus replicates in newborn and juvenile C57BL/6 J mice, causing lethal infection in neonates [13]. Evidence of zoonotic exposure also exists, with neutralizing antibodies against MKWV in Yezo deer and raccoons in Hokkaido, Japan [16].

Current models for studying such viruses face significant limitations. Traditional two-dimensional (2D) cell cultures cannot replicate the three-dimensional architecture, cellular diversity, and tissue-specific interactions of human organs, which are crucial for understanding viral pathogenesis [17,18]. Although animal models offer a systemic perspective, interspecies genetic differences often limit their translational relevance to human disease, alongside ethical and cost consideration [19]. In contrast, three-dimensional (3D) engineered tissues, which recapitulate key structural, and microenvironmental features of human organs, provide a more physiologyically relevant platform. These advanced in vitro systems enable more accurate study of viral replication, tissue-specific pathogenicity, and host responses, bridging the gap between conventional cell culture and animal models [20,21].

In this study, we isolated a novel MKWV strain (HLJ1) that showed limited replication in conventional mammalian cells and mouse models. However, this strain productively infected human 3D liver spheroids and rapidly adapted during serial passaging in this system. The resulting adapted strain exhibited enhanced replication efficiency in vitro and increased pathogenicity in vivo. Furthermore, molecular and serological surveillance of tick-bite patients in northeastern China revealed detectable MKWV RNA and specific antibodies, supporting the potential of this virus as an emerging tick-borne pathogen capable of infecting humans and other mammals.

## 2 Materials and methods

### 2.1 Sample collection

Ticks were collected in Heilongjiang Province, China, from May to July 2023 using a standard dragging method [22]. Species were identified via combined morphological and molecular techniques [23]. Ticks were pooled in groups of ten by species and collection site, and stored at −80 °C until processing.

Blood samples were collected in 2022 from patients presenting with tick bites at the First Hospital of Jilin University in 2022. A standardized questionnaire was used to record patient demographic information, medical history, and details of tick exposure.

### 2.2 Cells and mice

Mammalian cell lines, including Vero, BHK-21, DH82, MDBK, Huh-7, HepG-2, and SMMC-7721, were obtained from the National Collection of Authenticated Cell Cultures and maintained in Dulbecco's Modified Eagle Medium (DMEM) supplemented with 10% fetal bovine serum, 1% penicillin (10,000 units/ml) and streptomycin (10,000 μg/mL) at 37 °C with 5% CO_2_. Tick cells, including ISE6 from *Ixodes scapularis*, BME/CTVM23 from *Rhipicephalus microplus*, and IRE/CTVM19 from *Ixodes ricinus*, were sourced from the Tick Cell Biobank at the University of Liverpool, and cultured in Nunc™ Cell Culture Tubes (Thermofisher) using L-15 or L-15B300 culture medium at32 or 28 °C [[24], [25], [26]].

BALB/c, C57BL/6, and NYG mice were purchased from Changsheng biotechnology (Benxi, China). NYG mice, which lack mature T, B, and NK cells due to knock out of the *Prkdc* and *IL2RG* genes on a NOD background, were generated by the Animal Core Facility of Nanjing Medical University [27].

### 2.3 Human 3D liver spheroid construction

Human 3D liver spheroids were constructed via the NAC self-assembly strategy [[28], [29], [30]]. Primary human hepatocytes, liver sinusoidal endothelial cells, and Kupffer cells (IxCell Biotechnology) were mixed at specific ratios and co-incubated with NAC-Linker A and B (Puheng Biomedicine, NAC001) to facilitate NAC structure formation on the cell surfaces. The cell suspension was then seeded into 96-well 3D culture plates (Puheng Biomedicine, S-9601) at a density of 3000 cells/well. Plates were incubated at 37 °C with 5% CO₂ for 24 h to allow spheroid formation and stabilization. All experiments involving human 3D liver spheroids were approved by the Ethics Committee of the First Hospital of Jilin University (Approval No. 2024–810).

### 2.4 Virus isolation and titration

MKWV-positive tick homogenates were clarified by centrifugation at 12,000 rpm for 30 min. The supernatant was inoculated onto mammalian and tick cell monolayers for 2 h, after which the inoculum was replaced with fresh medium. Cells were monitored daily for CPE through three blind passages before culture harvest for MKWV detection.

Viral titers for the HLJ1 strain, quantified as the 50% tissue culture infectious dose w(TCID_50_), were determined in ISE6 cells using an immunofluorescence assay (IFA) endpoint and the Reed-Muench method [31]. The adapted NAC-Org5 strain was titrated on Vero cells using the same protocol.

### 2.5 Virus growth kinetics

Confluent monolayers of various cells (Vero, BHK-21, DH82, MDBK, SMMC-7721, Huh-7, HepG-2, ISE6, BME/CTVM23, and IRE/CTVM19) were infected with the HLJ1 or NAC-Org5 strain at a multiplicity of infection (MOI) of 0.1. After a 2-h adsorption period, cells were washed twice and replaced with fresh medium. Viral replication was monitored via RT-qPCR over 15 days. For spheroids, groups of five were infected at an MOI of 1 for 2 h, washed twice with Dulbecco's phosphate-buffered saline (D-PBS), and cultured in fresh medium.

### 2.6 Mouse infection experiments

Three-week-old male and female BALB/c, C57BL/6, and NYG mice were inoculated intraperitoneally(i.p.) with 4.00 × 10^5^ TCID_50_/mL. Tissues and blood were collected at 3, 7, 10, and 15 dpi. For the NAC-Org5 strain, three-week-old female BALB/c mice were inoculated i.p. with 4.00 × 10^5^ TCID_50_/mL with samples taken at 3, 5, 7, and 15 dpi. Suckling BALB/c mice were inoculated intracranially with either strain; samples were collected at 3, 5, and 7 dpi.

Mice were monitored for clinical signs throughout the experiment. Viral load in tissues was quantified by RT-qPCR. All animal experiments were approved by the Animal Welfare and Ethics Committee of the First Hospital of Jilin University (Approval No. 2023–0166) and conducted in compliance with the Regulations on the Administration of Laboratory Animals, with efforts to minimize suffering and animal numbers.

### 2.7 RNA extraction and PCR analysis

Viral RNA was extracted for tick homogenates, cell culture supernatants, mouse tissues, spheroids, and human serum using the TIANamp Virus RNA kit (TIANGEN) according to the manufacturer's instructions. cDNA was synthesized using the PrimerScript™ RT reagent Kit with gDNA Eraser (TaKaRa). For detection, quantitative PCR (qPCR) was performed using Premix Ex Taq™ (TaKaRa) in a 20 μL reaction consisting of 10 μL Premix Ex Taq, 0.4 μL each of forward and reverse primers (10 μM; Table S1), 0.8 μL probe, 1 μL cDNA, 0.4 μL ROX dye, and nuclease-free water. Cycling conditions were: 95 °C for 30 s, followed by 50 cycles of 60 °C for 5 s, 95 °C for 30 s. A threshold (Ct) < 40 was considered positivity. For complete genome sequencing, PCR was performed using Platinum™ SuperFi II DNA Polymerase (Thermo) with strain-specific primers (Table S1) in a semi-nested PCR protocol. First-round conditions: 98 °C for 30 s; 40 cycles of 98 °C for 30 s, 60 °C for 30 s, and 72 °C for 60 s; final extension at 72 °C for 5 min. Second-round PCR used the first-round product as template, under identical conditions. Amplicons were sequenced via the Sanger method (Sangon Biotech, Shanghai, China).

### 2.8 Electron microscopy

For negative staining, supernatant from MKWV-infected ISE6 cells was ultracentrifuged at 100,000 ×g for 2.5 h at 4 °C. The pellet was resuspended in 100 μL deionized water, adsorbed onto collodion‑carbon-coated copper grids, and stained with 2% phosphotungstic acid. Samples were imaged using an H-7650 transmission electron microscope (TEM; Hitachi) at 100 kV. For ultrathin sections, spheroids were fixed in 2.5% glutaraldehyde, postfixed in 1% osmium tetroxide, dehydrated, and embedded in Quetol 812 resin. Sections were stained with uranyl acetate and lead citrate prior to TEM imaging.

### 2.9 Immunofluorescence and histological staining

For immunofluorescence analysis (IFA), spheroid sections were permeabilized, blocked, and incubated overnight at 4 °C with primary antibodies: anti-MKWV NP protein (1:200), CD31 (Abcam, ab182981, 1:100), CD68 (Abcam, ab303565, 1:100), and ALB (BETHYL, A80-129P, 1:200). After washing, sections were incubated with fluorescent secondary antibodies (Cy3 goat anti-rabbit IgG for MKWV; Alexa Fluor 488 goat anti-mouse IgG for others) at 1:100 dilution for 1 h at room temperature. Nuclei were counterstained with DAPI. For histopathology, spheroid and mouse liver sections were stained with hematoxylin and eosin (H&E), dehydrated, cleared, and mounted for bright-field microscopy.

### 2.10 Spheroid functional assays

Spheroids and their conditioned media were harvested at 3 dpi. Cell viability was assessed using the Cell Counting-Lite® 3D Luminescent Cell Viability Assay kit (Vazyme). Culture medium was analyzed for alanine aminotransferase (ALT), aspartate aminotransferase (AST), and lactate dehydrogenase (LDH) using the commercial kits (Nanjing Jiancheng Bioengineering Institute, China; Dojindo Laboratories, Japan). Albumin (ALB) and urea were quantified with the Human Serum Albumin ELISA Kit (4 A BIOTECH, China) and Urea Assay Kit E2020 (Applygen Technologies, China), respectively [32,33].

### 2.11 Inflammatory cytokine analysis

Total RNA was extracted from spheroids (72 hpi) using the RNA-easy Isolation Reagent (Vazyme, China) and reverse transcribed (PrimeScript™ RT reagent Kit with gDNA Eraser,TaKaRa, Japan). qPCR was conducted with TB Green® Premix Ex Taq™ II (TaKaRa, Japan) under the following conditions: 95 °C for 30 s; 40 cycles of 95 °C for 5 s and 60 °C for 30 s. Primers are listed in Table S1. Reaction was run in triplicate. β-actin served as the reference gene. Relative expression of IL-1β, IL-6, IL-8, and TNFα was calculated using the 2^^-ΔCt^ method.

### 2.12 Cleaved Caspase-3 detection

Spheroid protein lysates (48 and 96 hpi) were prepared using RIPA lysis buffer with 1% PMSF, homogenized, and centrifuged. The proteins were denatured, separated by SDS–PAGE, and transferred to PVDF membranes. After blocking with 5% non-fat milk, membranes were incubated with anti- Caspase-3 antibody (Cell Signaling Technology, 144220, 1:1000) followed by HRP-conjugated secondary antibody. Signals were detected by enhanced chemiluminescence (ECL) and visualized with an imaging system (VILBER).

### 2.13 Phylogenetic analysis

Sequence alignment was performed with ClustalW using reference strains (Table S2). Maximum-likelihood phylogenetic trees were constructed in MEGA version 7.0 with 1000 bootstrap replicates; values >70 are shown [34].

### 2.14 Serological assays (ELISA and VNT)

MKWV-specific IgG/IgM were detected by indirect ELISA [35]. Recombinant MKWV nucleoprotein (Detai Bio, Nanjing, China) was coated (0.2 μg/well) overnight at 4 °C in 96-well plates. After blocking with 5% skim milk. Subsequently, diluted human serum (1:20) was added and incubated at 37 °C for 1 h. Following washes, horseradish peroxidase-conjugated rabbit anti-human IgG/IgM antibodies (Abcam, 1:20,000) was added. The reaction was developed with TMB substrate, stopped with 2 M H_2_SO_4_, and read using a spectrometer (ELx800; BioTek, Winooski, USA) at 450 nm. The cut-off was determined as the mean optical density of ten negative control serums plus 3 standard deviations. An ELISA index (OD sample/OD cut-off value) ≥ 1.2 was considered positivity.

For the viral neutralization test (VNT), serially diluted IgG-positive sera (starting at a dilution of 1:20) were mixed with 100 focus-forming units (FFU) of MKWV, incubated at 37 °C for 1 h, and added to Vero monolayers. After 5 days, infection was evaluated by IFA. The neutralization titer was the reciprocal of the highest dilution of reducing foci by ≥50% [35].

### 2.15 Statistical analysis

Data were analyzed in GraphPad Prism 9 software using Student's unpaired *t*-test. Results were presented as means ± standard deviation (SD). *p* < 0.05 was considered statistically significant (* *p* < 0.05, ** *p* < 0.01, and *** *p* < 0.001).

## 3 Results

### 3.1 Isolation and characterization of MKWV strain HLJ1 from ticks

**Fig. 1:**
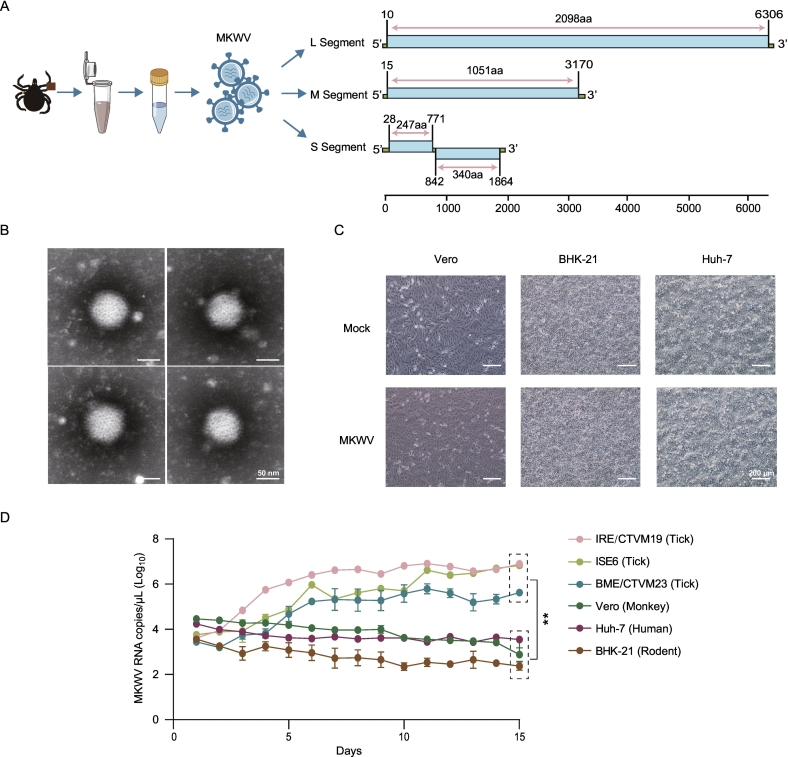
Isolation and characterization of the MKWV strain HLJ1. (A) Schematic representation of MKWV strain HLJ1 and its genomic organization. (B) Transmission electron micrograph of purified MKWV virions from the supernatant of ISE6 tick cells. Scale bars, 50 nm. (C) Bright-field microscopy of Vero, BHK-21 and Huh-7 cells inoculated with the HLJ1 strain, showing an absence of cytopathic effect. Scale bars, 200 nm. (D) Growth kinetics of the HLJ1 strain in mammalian (Vero, Huh-7 and BHK-21) cells, and tick-derived (IRE/CTVM19, ISE6, and BME/CTVM23) cells. Viral RNA in supernatants was quantified daily by RT-qPCR for 15 days post inoculation (dpi). Data are presented as mean ± SD; ** *p* < 0.01.

The MKWV strain HLJ1 was identified in *Ixodes persulcatus* ticks collected in Heilongjiang Province, China, with an overall detection rate of 1.92% (Table S3). Genomic characterization confirmed HLJ1 as a segmented RNA virus containing the characteristic L, M, and S segments, encoding the RNA-dependent RNA polymerase (RdRp), glycoprotein precursor, and nucleoprotein/non-structural protein, respectively (Fig. 1A). Comparative genomic analysis revealed that the HLJ1 strain shares 88.5–100% nucleotide and 90.3–100% amino acid identity with other reported MKWV strains (Tables S4-S7). Phylogenetic trees constructed from the L, M, and S segment sequences placed HLJ1 within a clade containing the Japanese strain MKW73 and related tick-borne phleboviruses, including Kuriyama virus, Mudanjiang phlebovirus, Qingdao tick phlebovirus, and Alxa tick phlebovirus, distinctly separate from mosquito- and sandfly-borne phleboviruses (Fig. S1-S3).

To specifically isolate MKWV, tick homogenates were screened using a semi-nested PCR protocol designed to exclude other common tick-borne viruses (tick-borne encephalitis virus, SFTSV, ALSV, BJNV, Nuomin virus). PCR-positive samples were centrifuged, filtered, and inoculated into ISE6 tick cells. Following three blind passages, the HLJ1 strain was successfully isolated, demonstrating robust replication in this cell line. Transmission electron microscopy of purified virions from ISE6 supernatants revealed enveloped, spherical particles approximately 100 nm in diameter, morphologically similar to SFTSV (Fig. 1B) [36]. In contrast, inoculation of various mammalian cell lines (Vero, BHK-21, Huh-7) failed to yield productive infection, with no cytopathic effect (CPE) observed in any tested line (Fig. 1C).

To specifically isolate MKWV, tick homogenates were screened using semi-nested PCR protocol designed to exclude other common tick-borne viruses, such as tick-borne encephalitis virus, SFTSV, ALSV, BJNV, and Nuomin virus. PCR-positive samples were centrifuged, filtered, and inoculated into ISE6 cells. Following three blind passages, the HLJ1 strain was successfully isolated, demonstrating robust replication in this cell line. Transmission electron microscopy of purified virions from ISE6 supernatants revealed enveloped, spherical particles approximately 100 nm in diameter, morphologically similar to SFTSV (Fig. 1B) [36]. In contrast, inoculation of various mammalian cell lines (Vero, BHK-21, and Huh-7) failed to yield productive infection, with no cytopathic effects (CPE) observed in any tested line (Fig. 1C).

### 3.2 HLJ1 strain exhibits limited replication in mammalian systems but efficient growth in tick cells

To evaluate its infectivity, we first assessed the growth kinetics of the HLJ1 strain in traditional two-dimensional (2D) cell lines. The strain replicated efficiently in tick-derived cells, particularly in ISE6 and IRE/CTVM19 cells, reaching titers of approximately 6.31 × 10^6^ copies/μL by 15 dpi. Viral loads were lower in BME/CTVM23 cells (∼3.98 × 10^5^ copies/μL). In stark contrast, replication in all tested mammalian cell lines was ineffective, with viral loads declining throughout the observation period (Fig. 1D).

We next investigated the pathogenicity of HLJ1 in vivo using murine models [37]. Intraperitoneal inoculation of three-week-old BALB/c and C57BL/6 mice (4.00 × 10^5^ TCID_50_) did not cause mortality or significant weight loss over 15 days. In female BALB/c mice, viral RNA (∼10^4^ copies/mg) was detected at 3 dpi in the heart, liver, spleen, and kidney, but largely cleared by 7 dpi, persisting only in the lungs at low levels (∼7.90 × 10^3^ copies/mg). Male BALB/c mice showed a more restricted and lower-titer infection, with RNA detectable only in the heart, liver, kidney, serum, and testes at 3 dpi (all <10^4^ copies/mg) (Fig. S4A). C57BL/6 mice were more resistant, with viral RNA found only in the spleen of a single female at 3 dpi (Fig. S4B).

To determine if immune deficiency facilitated infection, we challenged NYG mice (lacking T, B, and NK cells). While viremia was detected in all NYG mice at 3 dpi, overall pathogenicity and viral persistence were not markedly enhanced compared to wild-type strains. In female NYG mice, viral RNA was detected in the kidney (∼2.50 × 10^4^ copies/mg) and liver (∼ 5.01 × 10^4^ copies/mg) at 3 dpi, with low-level liver infection persisting to 7 dpi. Male NYG mice showed inconsistent distribution, with RNA detected only in the testes at 3 dpi (Fig. S4C).

Given the attenuated phenotype in juvenile and adult mice, we intracerebrally inoculated 3-day-old suckling BALB/c mice. This route did not cause mortality or weight loss. The highest viral load was detected in brain tissue at 7 dpi (∼ 5.01 × 10^8^ copies/mL), followed by the liver (∼ 5.01 × 10^7^ copies/mg) (Fig. S5A). Serial passaging of brain homogenates in neonates led to diminishing returns; by the third passage, viral titers in brain tissue approached the detection limit and were undetectable in other organs (Fig. S5B—C).

### 3.3 Human 3D liver spheroids support productive infection and adaptation of the HLJ1 strain

**Fig. 2:**
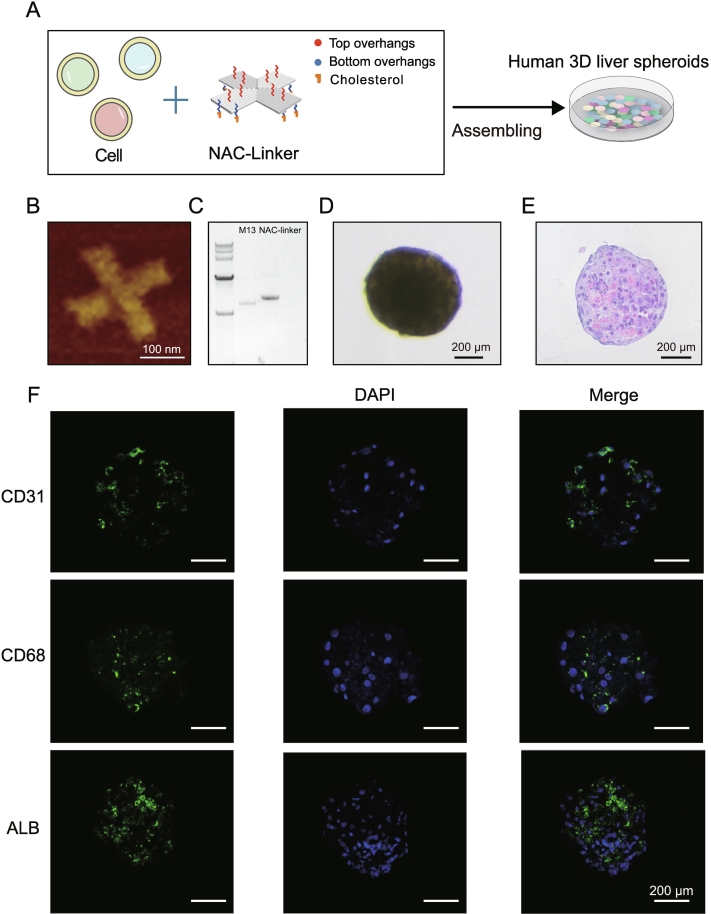
Assembly and characterization of human 3D liver spheroids via DNA origami NAC-linkers. (A) Schematic of 3D liver spheroid self-assembly from primary human hepatocytes, liver sinusoidal endothelial cells, and Kupffer cells using NAC-linkers. (B) Atomic force microscopy image of NAC-linkers. Scale bars, 200 nm. (C) 1% agarose gel electrophoresis confirming cholesterol-modified NAC-linkers assembly (lanes: DNA marker, M13mp18 scaffold, and NAC-linkers). (D) Bright-field image of a mature spheroid. (E) Hematoxylin and eosin (H&E) staining of a spheroid section. (F) Immunofluorescence staining of cell type markers in human 3D liver spheroids: albumin (ALB, hepatocytes), CD31 (endothelial cells), and CD68 (Kupffer cells). Scale bars, 200 μm.

Prompted by the reported isolation of the related MKW73 strain in human hepatoma cells [13] and the hepatic tropism of HLJ1 in mice, we employed a human 3D liver spheroid model to overcome the strain's recalcitrance in standard mammalian systems. Spheroids were generated via a scaffold-free DNA origami technique, using NAC-linkers to drive the rapid self-assembly of primary human hepatocytes, liver sinusoidal endothelial cells, and Kupffer cells into physiologically relevant 3D structures within 24 h (Fig. 2A-F, Tables S8-S10). This system preserves long-term liver function and provides a human-relevant platform for pathogenicity assessment [30].

**Fig. 3:**
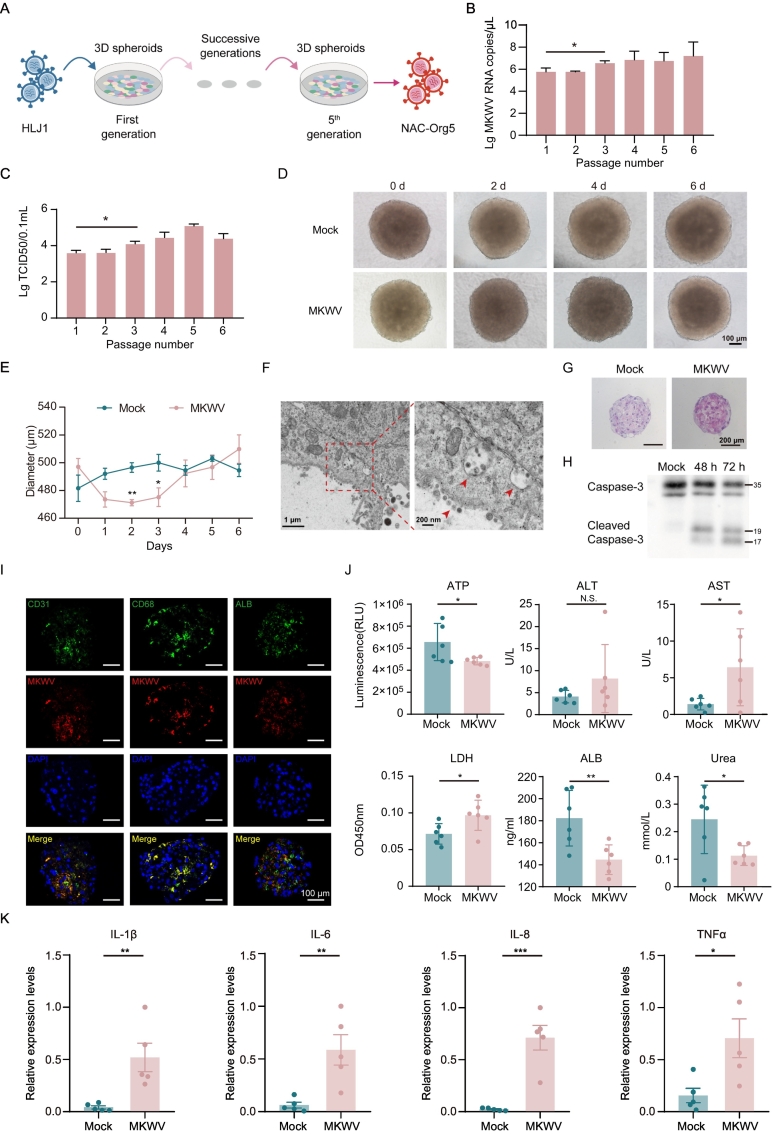
Adaptation and pathogenesis of MKWV in human 3D liver spheroids. (A) Schematic of serial passaging of the HLJ1 strain in spheroids, yielding the adapted NAC-Org5 strain. (B, C) Viral RNA copies (B) and TCID₅₀ titers (C) across passages (P1-P5). (D) Bright-field image of spheroids infected with passage 5 (P5) virus, showing structural disruption. Scale bar, 100 μm. (E) Quantification of spheroid diameter post-infection. (F) Transmission electron micrographs of virions within cytoplasmic vesicles of infected spheroids. Scale bars: 1 μm (left), 200 nm (right). (G) Representative images and quantification of nuclei showing infection-induced cell death. Scale bar, 200 μm. (H) Western blot detecting cleaved caspase-3 in spheroids at 48 and 72 h post-infection (hpi). (I) Multiplex immunofluorescence showing NAC-Org5 tropism for CD31^+^ endothelial cells and CD68^+^ Kupffer cells, with weaker detection in ALB^+^ hepatocytes. Scale bar, 200 μm. (J) Functional assessment of infected spheroids: ATP (viability), ALT/AST/LDH (damage), ALB/urea (synthetic function). (K) RT-qPCR analysis of pro-inflammatory cytokine mRNA expression, normalized to β-actin. Data are mean ± SD (*n* = 5 biological replicates). *p < 0.05, **p < 0.01.

Inoculation of HLJ1 into these spheroids resulted in robust viral replication, with titers reaching approximately 3.16 × 10^7^ copies/mL at 6 dpi. Serial passaging of the virus within spheroids significantly increased viral titers by passage 3 (P3), which were sustained in subsequent passages (Fig. 3A-C). Infection caused severe structural disruption, including reduced spheroid diameter and irregular morphology (Fig. 3D-E). Transmission electron microscopy confirmed viral replication, showing virions within cellular membranes and cytoplasmic vesicles (Fig. 3F). The adapted virus from passage 5, designated NAC-Org5, was used for all subsequent characterization. Infection with NAC-Org5 induced significant cell death, evidenced by an approximately 12.7% reduction in nuclear count (Fig. 3G) and the cleavage of caspase-3, confirming apoptosis activation (Fig. 3H). Immunofluorescence staining revealed robust colocalization of viral antigen with CD31^+^ and CD68^+^ cells, indicating strong tropism for sinusoidal endothelial cells and Kupffer cells, respectively. In contrast, signal in ALB^+^ hepatocytes was markedly weaker (Fig. 3I).

Functional assays demonstrated that infection impaired spheroid viability and synthetic capacity. A significant decrease in cellular ATP levels was observed alongside marked elevations in aspartate aminotransferase (AST) and lactate dehydrogenase (LDH), indicating cell damage and death. Furthermore, levels of key liver synthesis products, albumin (ALB) and urea, were significantly reduced (Fig. 3J). Concurrently, infection triggered a pronounced pro-inflammatory response, with mRNA levels of IL-1β, IL-6, IL-8, and TNF-α significantly upregulated (Fig. 3K). Collectively, these results demonstrate that the human 3D liver spheroid model enables productive infection by the otherwise refractory HLJ1 strain, facilitates viral adaptation, and reveals a pathology characterized by cell death, functional impairment, and a robust inflammatory response.

### 3.4 Adaptation of MKWV in human 3D liver spheroids confers enhanced mammalian virulence

**Fig. 4:**
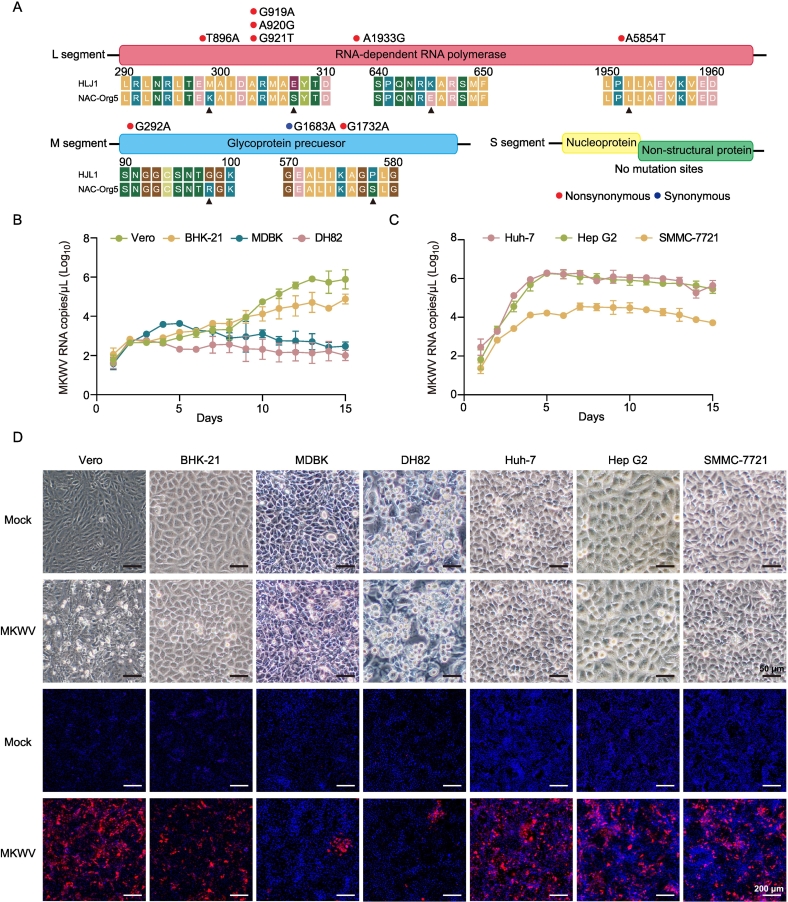
MKWV strain NAC-Org5 exhibits cellular infectivity. (A) Analysis of mutations in NAC-Org5 in comparison to the parental strain HLJ1. (B) Growth kinetics of NAC-Org5 strain in Vero, BHK-21, MDBK, and DH82 cells. (C) Growth kinetics of NAC-Org5 strain in human liver cells, including Huh-7, HepG-2, and SMMC-7721 cells. (D) Infection of the NAC-Org5 strain in multiple mammalian cell lines observed by bright-field microscopy (BF) and immunofluorescence assay (IFA). Scale bars, 50 μm for BF and 200 μm for IFA.

To define the genetic basis for the increased virulence of the NAC-Org5 strain, we sequenced its complete genome. Comparative analysis with the parental HLJ1 strain, the NAC-Org5 strain contained 9 nucleotide mutations distributed across the L and M segments revealed nine nucleotide mutations across the L and M segments, with no changes in the S segment. Six non-synonymous mutations in the L segment (T896A, G919A, A920G, G921T, A1933G, and A5647T) resulted in amino acid substitutions at positions 299 (M → K), 307 (E → S), and 1952 (I → L). In the M segment, two non-synonymous mutations (G292A, G1732A) led to substitutions at positions 98 (G → R) and 578 (P → S), alongside one synonymous change (G1683A) (Fig. 4A).

We next assessed whether these mutations altered viral tropism and replicative capacity. The NAC-Org5 strain exhibited significantly enhanced replication in mammalian cells compared to HLJ1. In Vero and BHK-21 cells, viral loads increased steadily, peaking at ∼6.31 × 10^5^ and ∼ 7.94 × 10^4^ copies/μL, respectively, by 15 dpi. Modest replication was observed in MDBK cells, while proliferation in DH82 cells was limited (Fig. 4B). Notably, the strain replicated most efficiently in human hepatocellular carcinoma cell lines (Huh-7 and HepG-2), reaching titers of ∼1.99 × 10^6^ copies/μL (Fig. 4C). This human-cell preference suggests adaptation acquired during serial passage in liver spheroids.

Infection with the NAC-Org5 strain induced distinct CPE, characterized by cell rounding, shrinkage, rounding, and detachment, in Vero cells by 7dpi (Fig. 4D). Immunofluorescence assays confirmed abundant viral antigens in Vero, BHK-21, and human hepatoma cells (Huh-7, HepG-2, and SMMC-7721), correlating with RT-qPCR data and indicating broadened mammalian cell tropism (Fig. 4D). Strikingly, this enhanced phenotype was specific to mammalian systems; replication in tick-derived cell lines (ISE6, BME/CTVM23, IRE/CTVM19) was not improved relative to the parental HLJ1 strain (Fig. S6). This suggests the adaptive mutations confer a selective advantage in mammalian cells without altering fitness in the arthropod vectors highlighting a fundamental shift in host-specific adaptation.

**Fig. 5:**
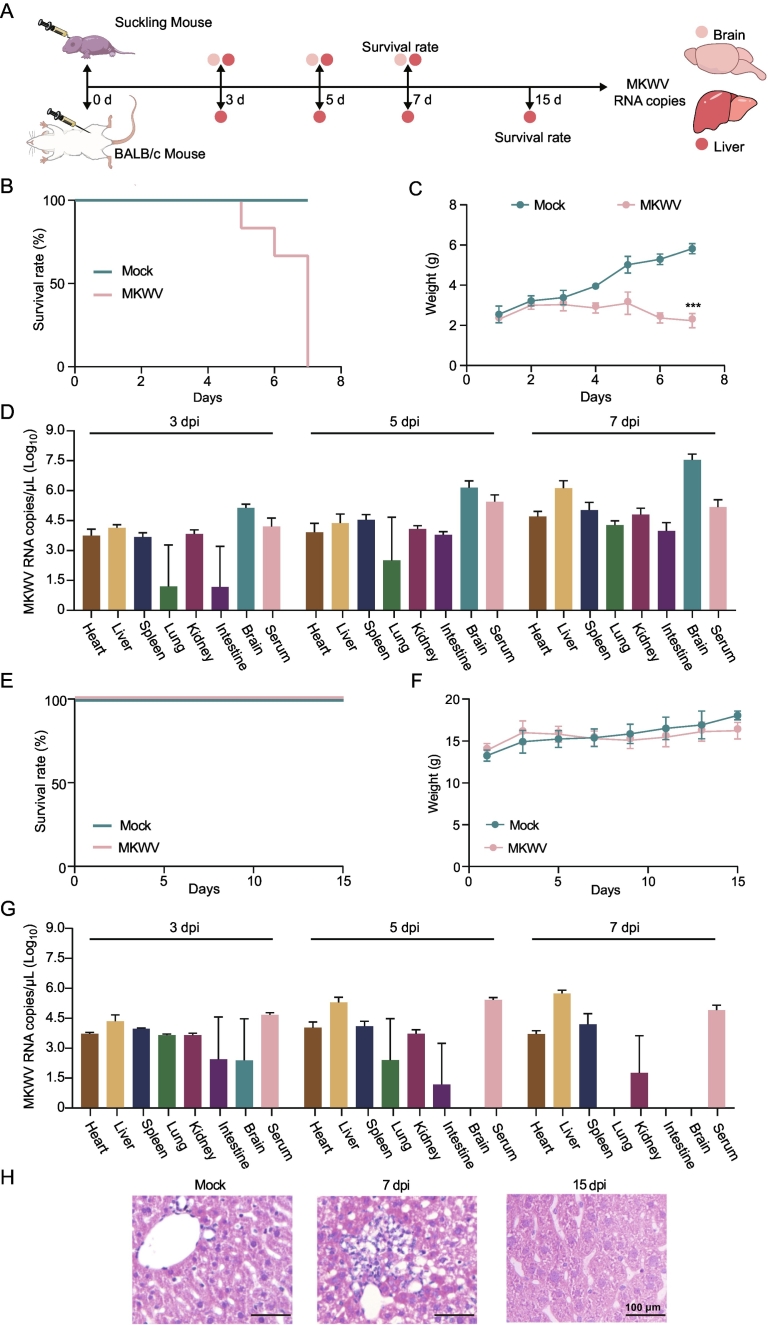
Pathogenicity of the NAC-Org5 strain in murine models. (A) Experimental schematic for intracranial (3-day-old) and intraperitoneal (3-week-old) inoculation of BALB/c mice. (B, C) Survival (B) and weight change (C) of suckling mice after NAC-Org5 infection. (D) Viral load in tissues and blood of suckling mice at 7 dpi. (E, F) Survival (E) and weight change (F) of 3-week-old mice. (G) Viral load in tissues and blood of 3-week-old mice at 7 dpi. Data are from 3 independent experiments. (H) Representative H&*E*-stained liver sections from 3-week-old mice at 7 and 15 dpi, showing inflammatory infiltrates and hepatocyte necrosis that resolves by 15 dpi. Scale bar, 100 μm. ***p < 0.001.

The expanded cell tropism translated to markedly increased pathogenicity in vivo. Intracranial inoculation of NAC-Org5 in suckling BALB/c mice caused 100% mortality (6/6) within 7 days, accompanied by significant weight loss (Fig. 5A-C). Viral loads in the brain and liver reached approximately 1.99 × 10^10^ and 2.51 × 10^8^ copies/mg, respectively (Fig. 5D). In 3-week-old mice, the infection was sublethal but persistent, with the liver remaining the primary site of replication (∼1.58 × 10^6^ copies/mg at 7 dpi) before clearance by 15 dpi (Fig. 5E-G). Histopathological analysis of livers from infected 3-week-old mice at 7 dpi revealed multifocal inflammatory infiltrates and hepatocyte necrosis. These lesions largely resolved by 15 dpi, consistent with viral clearance (Fig. 5H). The pronounced hepatic tropism and pathology mirror the damage observed in human 3D liver spheroids, confirming the physiological relevance of the model.

### 3.5 MKWV infection in patients with tick bites

The consistent liver pathology induced by the NAC-Org5 strain in human 3D liver spheroids and a murine model underscores a potential pathogenesis for MKWV. We therefore implemented active surveillance of hospitalized, tick-bitten patients in Jilin Province, northeastern China. Among 145 serum samples collected, MKWV was detected via RT-qPCR in two cases (1.4%), although the viral load was insufficient for genome sequencing. Serologic investigation revealed an MKWV-specific IgG positivity rate of 4.8% (7/145) and a neutralizing antibody rate of 3.4% (5/145); IgM antibodies were not detected in any sample (Table S11).

## 4 Discussion

In this study, we isolated the MKWV strain HLJ1 from *Ixodes persulcatus* ticks. This isolate demonstrated limited replication in standard mammalian cell lines and mouse models, yet it productively infected human 3D liver spheroids. Serial passaging within this system yielded a viral titer increase, suggesting adaptive evolution. The resulting adapted strain (NAC-Org5) exhibited enhanced virulence, inducing significant cytopathic effects in Vero cells, infecting a broad range of mammalian cells, and causing 100% mortality in suckling mice, while establishing a transient infection in three-week-old mice. Pathological analysis revealed liver inflammatory infiltration in mice, a phenotype mirrored in infected 3D liver spheroids, which displayed cell death and impaired synthetic function. This integrated approach, combining a physiologically relevant human in vitro model with in vivo validation, provides a novel framework for assessing the potential pathogenicity of emerging tick-borne viruses and underscores the latent risk of MKWV.

The advancement of 3D-engineered tissues offers a powerful tool for virology. Organoids and spheroids recapitulate key aspects of the human microenvironment, including cell-cell interactions and tissue architecture, which are often prerequisites for successful viral infection. For example, iPSC-derived liver organoids support efficient, long-term hepatitis B virus (HBV) replication and model virus-induced liver dysfunction more effectively than hepatocytes [38]. Similarly, adult stem cell-derived liver organoids sustain the complete HBV replication cycle [[39], [40], [41]]. Enhanced polarization and receptor expression in 3D-cultured Huh-7 cells also increase susceptibility to hepatitis C virus compared to traditional 2D monolayers [42,43]. Furthermore, 3D systems boost the yield of infectious particles for several animal viruses, including Suid herpesvirus 1 and bovine adenovirus, highlighting their potential as innovative platforms for virus isolation and study.

In an era of frequent viral public health emergencies, assessing the threat of emerging viruses or zoonotic viruses is urgent. Organ-specific models are increasingly used for this purpose. For instance, human respiratory organoids evaluate the zoonotic potential of avian swine influenza viruses [44], skin organoids model monkeypox virus infection for drug screening [45], and brain organoids elucidate Zika virus pathogenesis [46]. Currently, many novel tick-borne viruses are identified only through epidemiology, lacking functional models to gauge their public health significance. Organoids and spheroids could bridge this critical gap. While not perfect replicas of human organs, they represent the most physiologically relevant in vitro systems available. We constructed heterologous human 3D liver spheroids from primary hepatocytes, liver sinusoidal endothelial cells, and Kupffer cells from different donors. These spheroids maintained robust structural integrity and stability during long-term culture. Although it is unclear whether Kupffer cells recognize or selectively adhere to non-autologous cells within this system, existing literature supports their stable integration in mixed-donor environments [[47], [48], [49]]. This contrasts with their in vivo behavior under inflammatory conditions, where they can clear allogeneic cells, indicating that recognition is highly context-dependent [50]. Our findings suggest that in our optimized model, Kupffer cells establish stable interactions without overt immune incompatibility, though subtle donor-specific effects cannot be ruled out. A direct comparison of autologous versus heterologous spheroids will be necessary to precisely quantify the impact of donor origin on spheroid function and homeostasis.

Our study demonstrated that human 3D liver spheroids support efficient replication of the MKWV strain HLJ1, which could not be cultured in mammalian cell lines or effectively replicated in mice. Continuous culture in this system selected for the adaptive mutant strain NAC-Org5, which gained the ability to infect diverse mammalian cells, damage to liver spheroids, and cause liver-specific pathology in mice. Although immunofluorescence revealed predominant infection of non-parenchymal cells (Kupffer and endothelial cells), our methodology cannot rule out infection of other cell types at lower levels.

The hepatotropism appears to be a characteristic shared with other tick-borne phenuiviruses. For instance, SFTSV and GTV infections in mice induce significant hepatic lesions [11,51]. In contrast, the NAC-Org5 strain did not produce the splenic or renal pathology associated with these relatives. This restricted tropism may reflect a liver-specific adaptation acquired during organoid passaging, or it may result from the transient nature of infection, which limited the development of extrahepatic lesions. Furthermore, SFTSV infection in liver cells activates the NF-κB pathway and increases pro-inflammatory cytokines, a response mirrored in our NAC-Org-infected spheroids, suggesting conserved immune activation mechanisms [52].

The biological characteristics of NAC-Org5 strain differ notably from other reported MKWV strains, which show variable cell tropism (e.g., replication in Huh-7 cells and Vero cells) [13,15]. Despite these phenotypic differences, these strains share high amino acid identity (>98% in structural proteins), implying that a limited number of critical mutations can dramatically alter pathogenicity. Compared to the parental HLJ1, NAC-Org5 possesses only six amino acid substitutions, with four in the L segment and two in the M segment. The M segment mutations are of particular interest, as in bunyaviruses, the Gn protein facilitate receptor binding and endocytosis, while Gc mediates endosomal membrane fusion [[53], [54], [55]]. Future research must elucidate how the specific glycoprotein mutations contribute to the enhanced pathogenicity of the NAC-Org5 strain.

Infection outcomes were also influenced by host factors. Female BALB/c mice exhibited higher viral loads in the liver and blood than males, suggesting sexual dimorphism in viral replication. This may be driven by estrogen, which can modulate viral receptor expression and suppress type I interferon (IFN-α/β) signaling, thereby dampening the innate antiviral response [56,57]. Host age was equally critical: NAC-Org5 infection caused 100% mortality in suckling mice but was cleared efficiently by 3-week-old weanlings. This stark contrast is attributed to immune system maturation. Neonates possess underdeveloped innate immunity, including immature Kupffer and natural killer (NK) cell function, which weaken viral clearance and exacerbate tissue injury, a vulnerability also observed in neonatal Zika virus infection models [58]. In contrast, weanlings mount robust adaptive immunity, generating neutralizing antibodies and cytotoxic T lymphocytes (CTLs) that effectively control infection [59,60]. Employing immunodeficient mouse models (e.g., AG129 or STAT1^−/−^ mice) in future studies will help dissect the specific immune mechanisms governing MKWV pathogenesis [61,62].

Epidemiological surveys have detected MKWV in tick species, including *Haemaphysalis concinna*, *Ixodes persulcatus*, and *Dermacentor silvarum,* across northeastern China [[14], [15], [16]]. To investigate human exposure risk, we conducted molecular and serological testing on tick-bitten patients in Jilin Province. Seroprevalence was 4.8% for IgG antibodies, with five samples showing neutralizing activity. Furthermore, 1.4% of samples were positive for viral RNA. These findings indicate a tangible, though likely low, risk of human MKWV infection. However, reported serological cross-reactivity between MKWV and the closely-related Kuriyama virus in Japan suggests that seroprevalence may be overestimated [16]. This is particularly relevant given the co-circulation of the antigenically related Mudanjiang phlebovirus (MJPV) in the same region.

In summary, this study establishes a novel paradigm for assessing the public health risk of emerging tick-borne viruses by integrating human 3D spheroid model with epidemiological surveillance. Our findings highlight the utility of primary cell-derived 3D systems for studying fastidious pathogens and provide direct evidence suggesting MKWV as a potential contributor to emerging infectious diseases in northeastern China. Moving forward, it is essential to incorporate dedicated strategies for MKWV and related viruses into future research and public health agendas.

## Declaration of generative AI and AI assisted technologies in the writing process

During the preparation of this work, the author used DeepSeek to check for spelling and grammar errors. After using this tool, the author reviewed and edited the content as needed and takes full responsibility for the content of the published article.

## Ethics declarations

This study was approved by the Ethics Committee of the First Hospital of Jilin University. All patients provided written informed consent for the use of their samples and data. The animal experimental protocol was approved by the Institutional Animal Care and Use Committee of the First Hospital of Jilin University.

## CRediT authorship contribution statement

**Wenbo Xu:** Writing – original draft, Investigation. **Liyan Sui:** Writing – review & editing, Validation. **Nan Liu:** Writing – review & editing, Validation. **Lesley Bell-Sakyi:** Writing – review & editing, Resources. **Yicheng Zhao:** Writing – original draft, Conceptualization. **Yuanzhi Wang:** Methodology, Investigation. **Yinghua Zhao:** Investigation. **Changfeng Zhu:** Writing – review & editing, Resources. **Quan Liu:** Writing – review & editing, Funding acquisition, Conceptualization.

## Consent for publication

All authors contributed to the article and approved the final manuscript.

## Declaration of competing interest

The authors declare that they have no known competing financial interests or personal relationships that could have appeared to influence the work reported in this paper.

## Data Availability

All viral genome sequences obtained in this study have been deposited in GenBank and assigned accession numbers PQ215521 to PQ215523 for HLJ1 strain and PP999500 to PP999502 for NAC-Org5 strain.

## Appendix A Supplementary data

Supplementary material Image 1
