# Supplementary material for: Human 3D liver spheroids support productive infection of a novel tick-borne phenuivirus
Source: One Health. 2026 Jan 10;22:101321. doi: 10.1016/j.onehlt.2026.101321 (PMC12828834; doi:10.1016/j.onehlt.2026.101321)
Supplement: Supplementary file 1 — Supplementary material [file mmc1.docx]

Supplementary Material

**Human 3D liver spheroids** **support productive infection of a novel tick-borne phenuivirus**


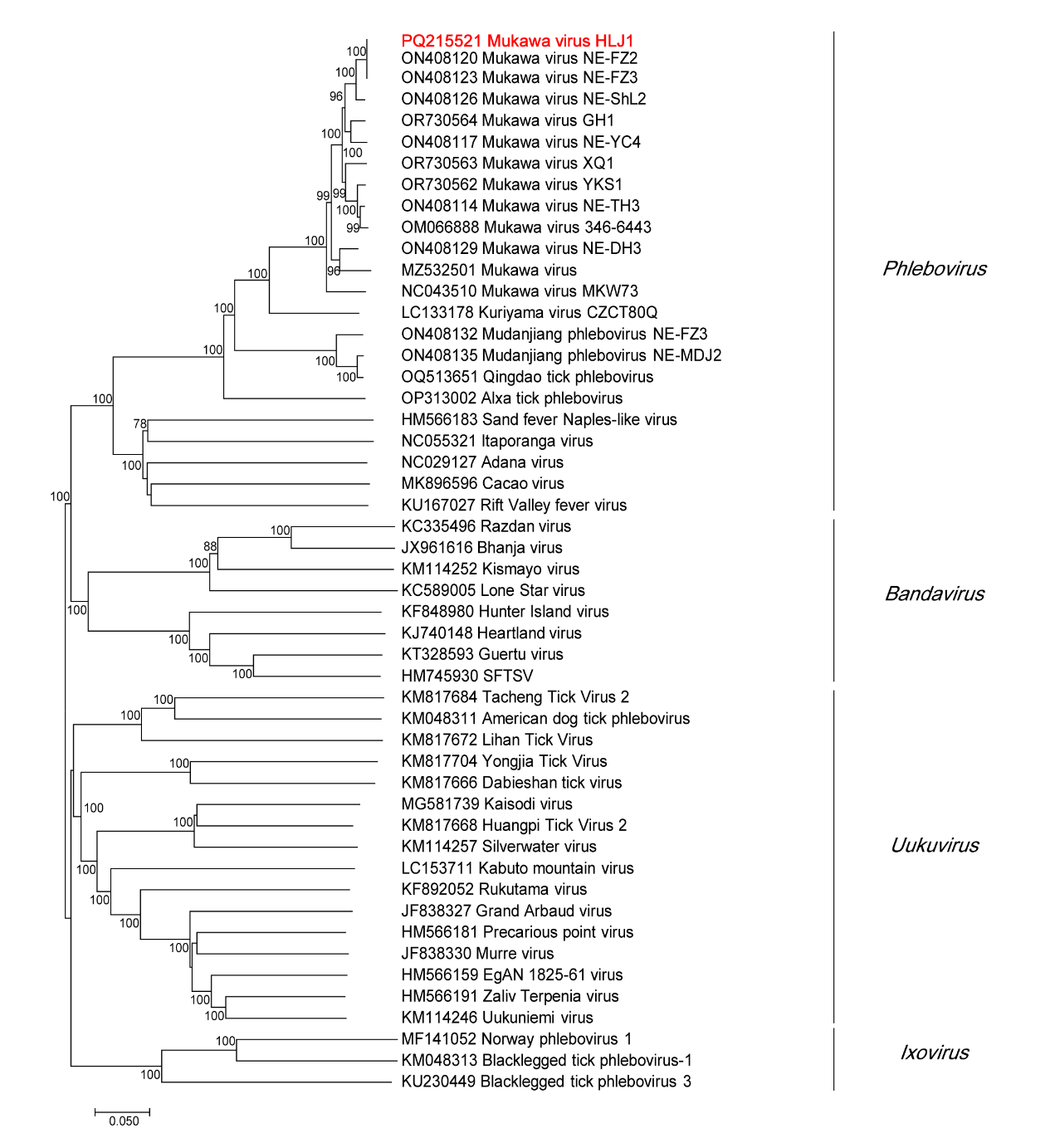


**Fig. S1. Phylogenetic analysis of the L segment of phenuiviruses.** The nucleotide sequences of the M segment of phenuiviruses were analyzed using the maximum likelihood method (ML) in the MEGA 7.0 software. Bootstrapping analysis was performed with 1,000 replicates, and values greater than 70 were considered significant and are displayed in the tree.

**
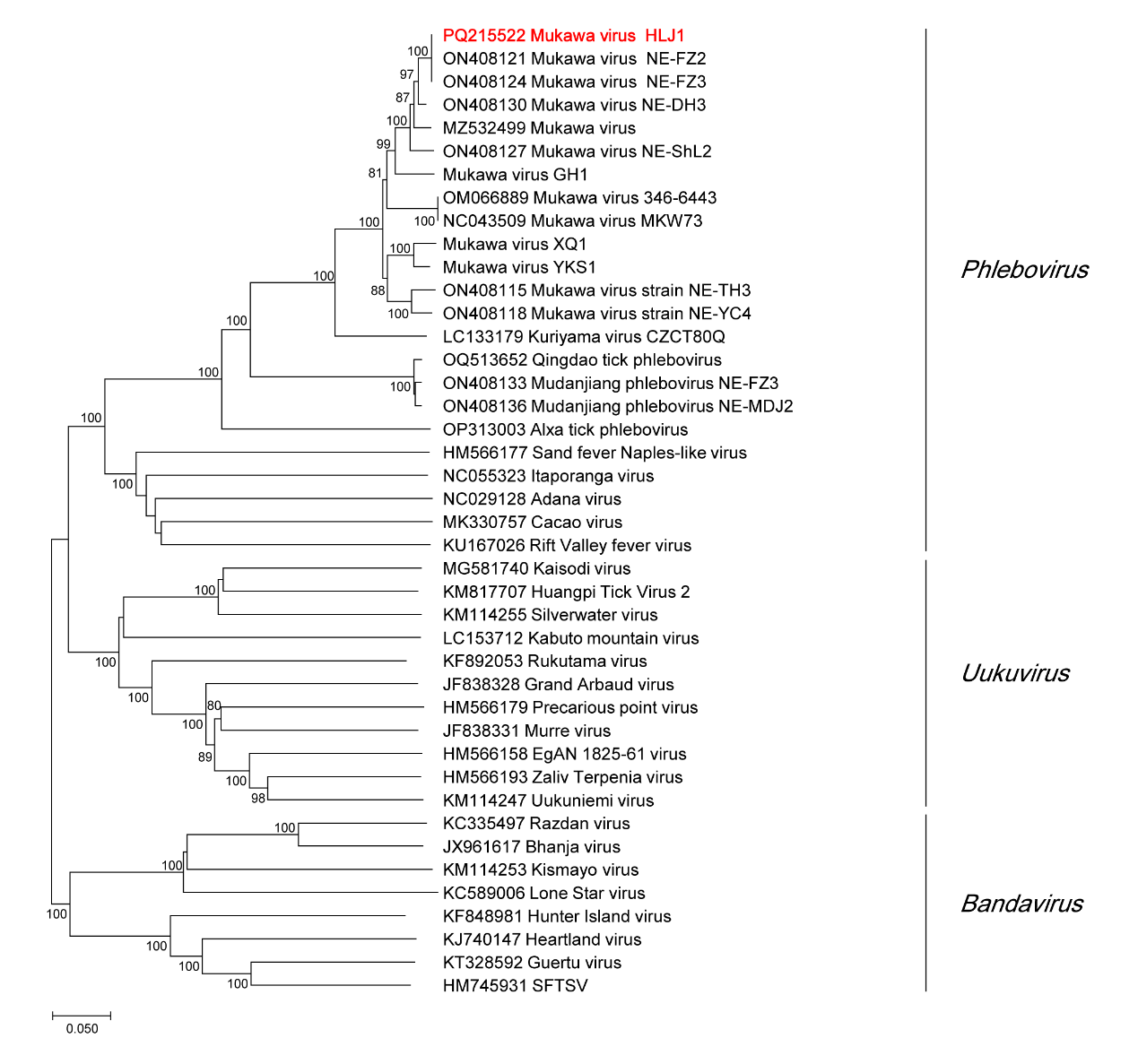
 Fig. S2. Phylogenetic analysis of the M segment of phenuiviruses.** The nucleotide sequences of the M segment of phenuiviruses were analyzed using the maximum likelihood method (ML) in the MEGA 7.0 software. Bootstrapping analysis was performed with 1,000 replicates, and values greater than 70 were considered significant and are displayed in the tree.

**
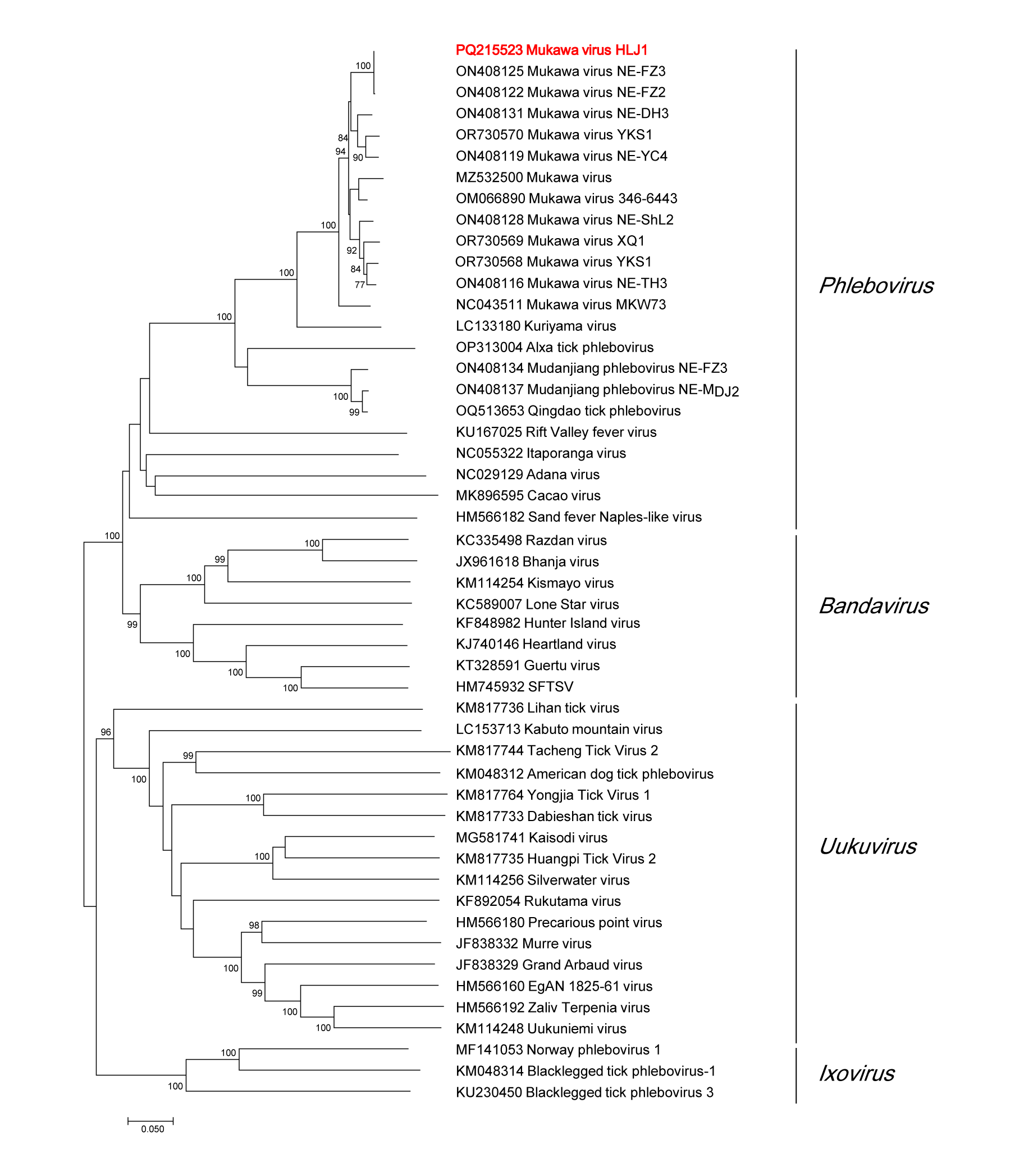
**

**Fig. S3. Phylogenetic analysis of the S segment of phenuiviruses.** The nucleotide sequences of the S segment of phenuiviruses were analyzed using the maximum likelihood method (ML) in the MEGA 7.0 software. Bootstrapping analysis was performed with 1,000 replicates, and values greater than 70 were considered significant and are displayed in the tree.


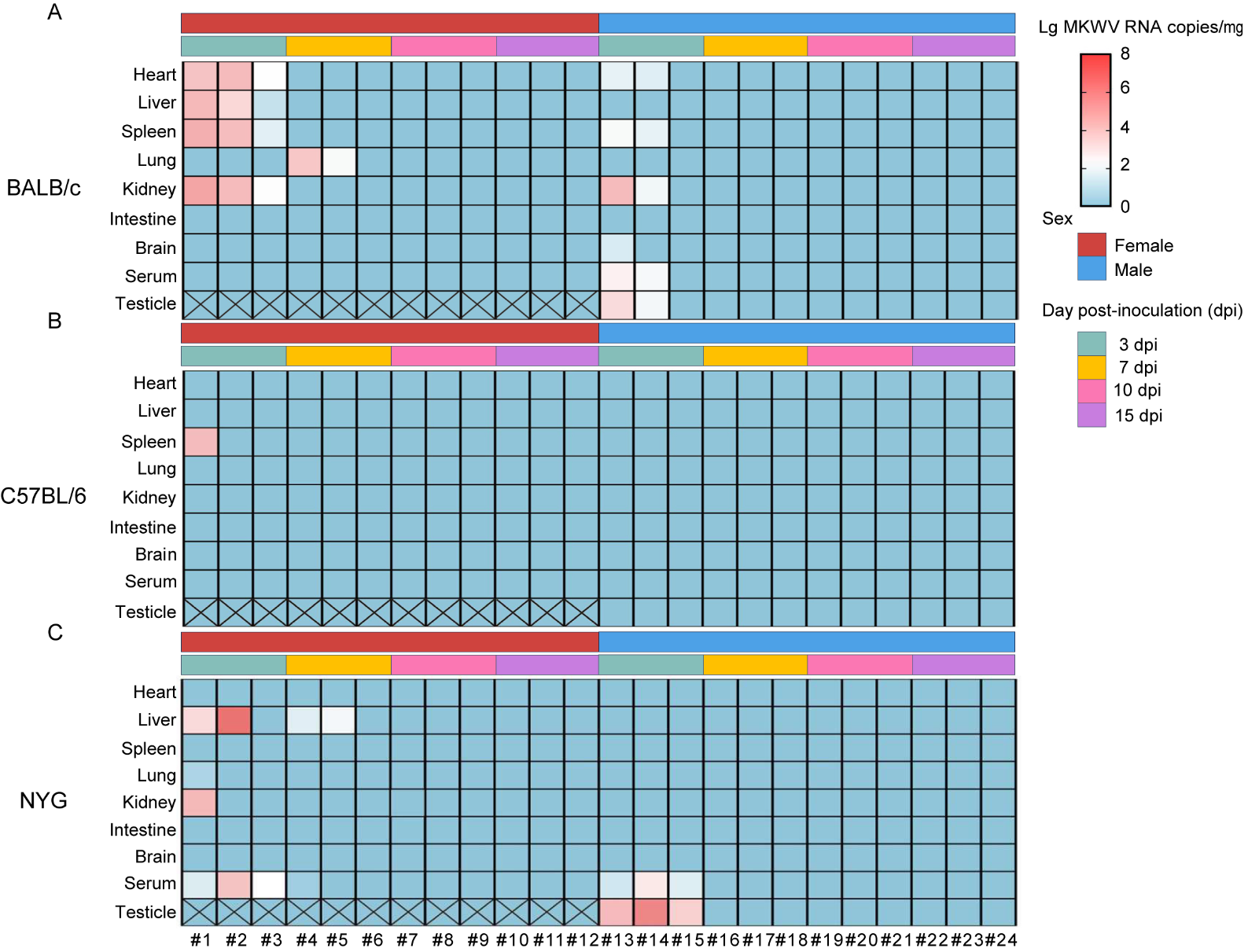


**Fig. S4. MKWV strain HLJ1 infection in different strains of mice.** (A) Viral RNA load in BALB/c inbred mice. (B) Viral RNA load in C57BL/6 inbred mice. (C) Viral RNA load in NYG mice. Tissues (heart, liver, spleen, lung, kidney, intestine, brain, and testicle) and serum samples were collected from three individuals in each group on 3, 7, 10, and 15 days post-inoculation.

**
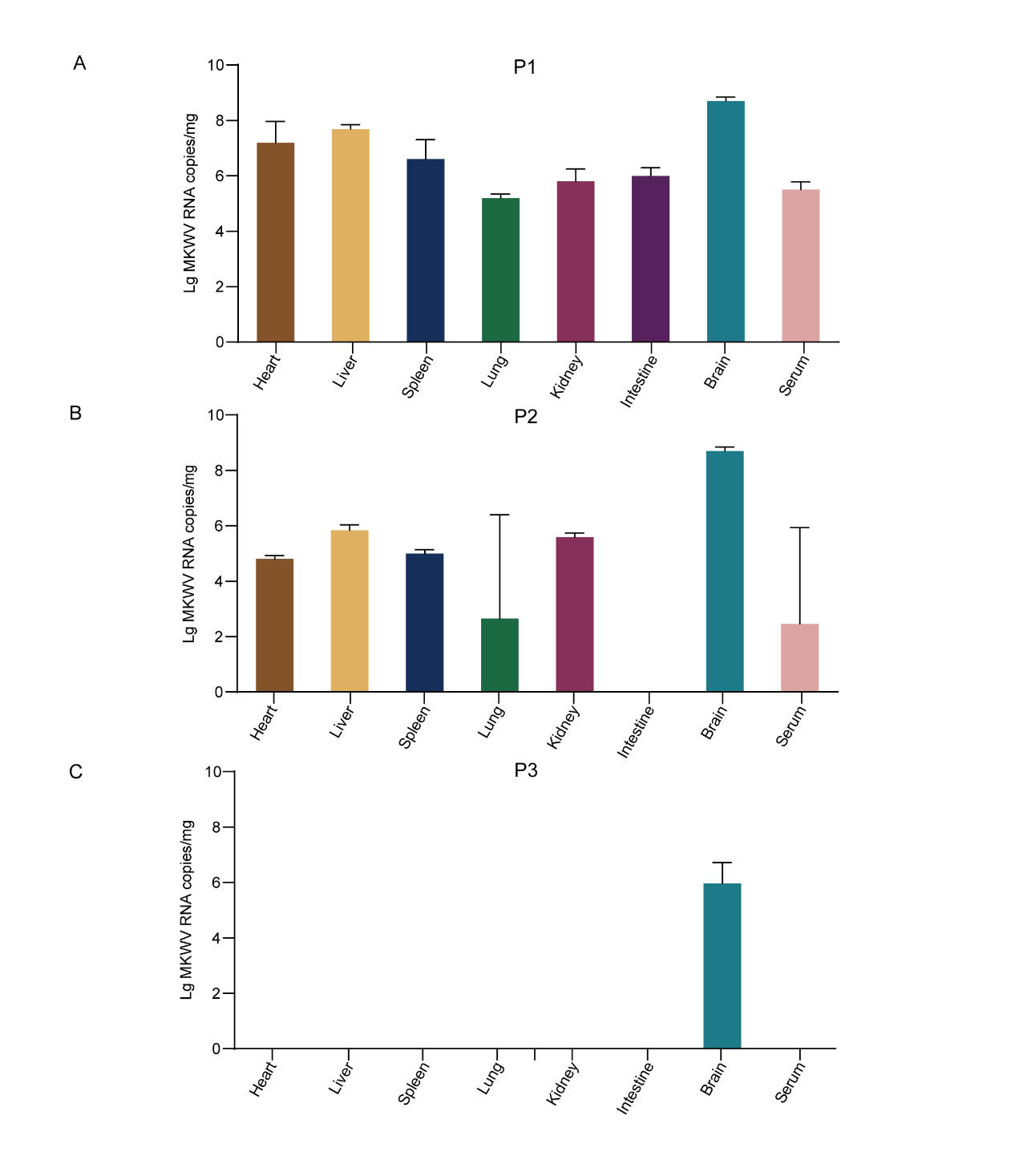
 Fig. S5. Serial passage of MKWV strain HLJ1 in BALB/c suckling mice.** The HLJ1 strain was inoculated into 3-day-old BALB/c suckling mice, and brain tissues were homogenized 7 days post-inoculation to prepare virus storage for subsequent passages. The suckling mice from the initial inoculation were defined as passage 1 (P1), and this process continued up to passage 3 (P3). Tissues (heart, liver, spleen, lung, kidney, intestine, brain, and testicle) and serum samples from P1 (A), P2 (B), and P3 (C) were collected at 7 dpi for RT-qPCR detection.


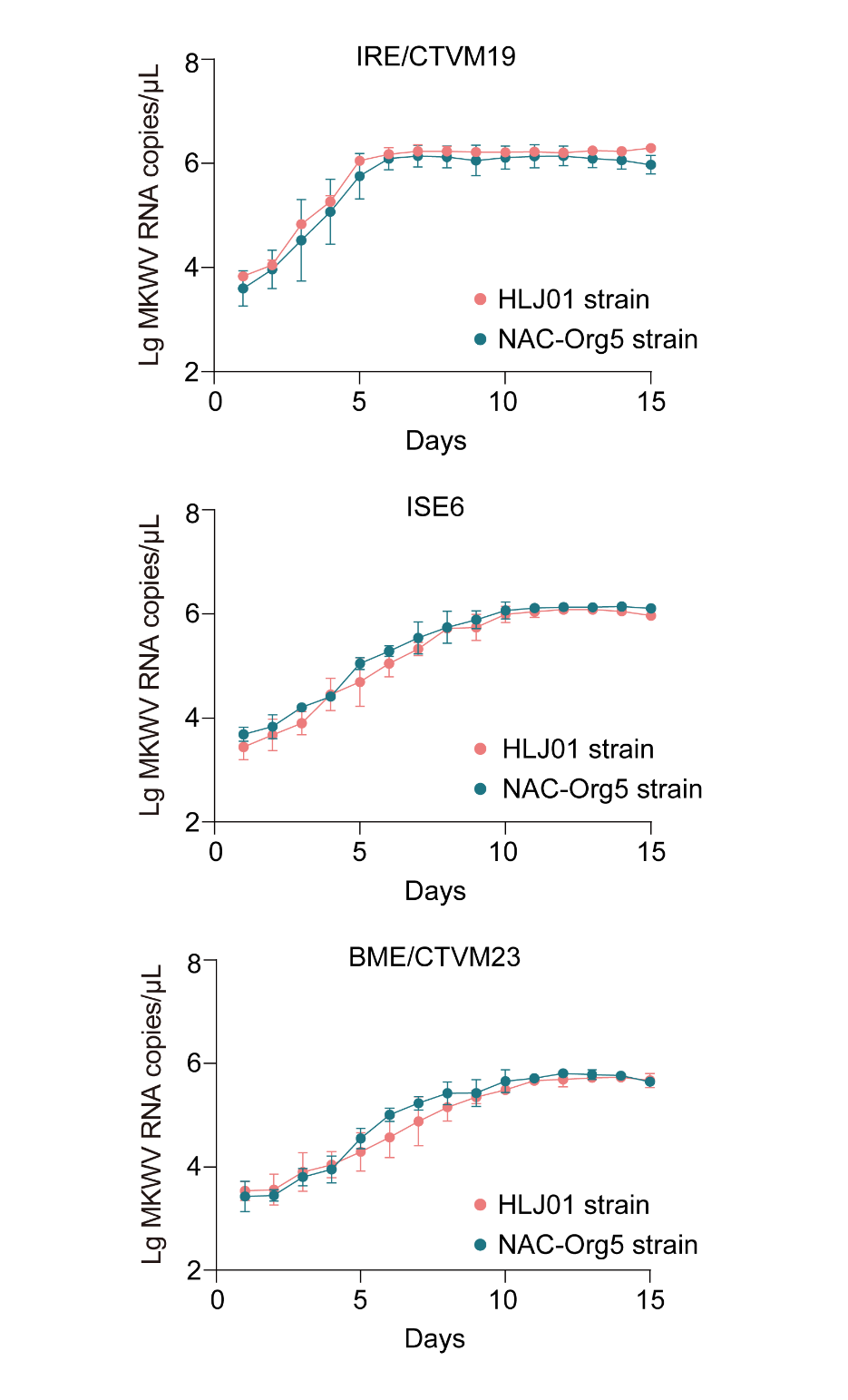
**Fig.** **S6. Growth kinetics of NAC-Org5 and HLJ1 strains in tick-derived cells.** NAC-Org5 and HLJ1 strains were inoculated into IRE/CTVM19, ISE6, and BME/CTVM23 cells. Cell culture supernatants were collected daily up to 15 days post-inoculation (dpi) and analyzed by RT-qPCR to quantify viral RNA levels.

**Table S1. Primers used in the present study.**

| Gene | Primer | Position (bp) | Sequence (5' to 3') | Polarity | Amplicon (bp) |
| --- | --- | --- | --- | --- | --- |
| Detection |  |  |  |  |  |
| S segment | MKWV- F | 500 | CCCTGAGCAGRGAYGATC | + | 151 |
|  | MKWV- R | 651 | CCTTGTCAGAGAAGAAGTTG | - |  |
|  | MKWV- Probe | 520 | ACTGCTCTSGTGGATGCTCA | + |  |
| Genome amplification |  |  |  |  |  |
| L segment | 1 MKWV F | 1 | CGCCCATTCATGGAACCAA | + | 893 |
|  | 1 MKWV R1 | 927 | ATCTGCCATCAGTGTACTCC | - |  |
|  | 1 MKWV R2 | 894 | ATCTATTGCCATCTCAGTCAGC | - |  |
|  | 2 MKWV F | 784 | GTGAACTCAGTTATAGCCCAA | + | 905 |
|  | 2 MKWV R1 | 1749 | CCAACATCCTCATAAGGCTTG | - |  |
|  | 2 MKWV R2 | 1689 | TTATCCAGGGCCATAGACACA | - |  |
|  | 3 MKWV F1 | 1651 | CTCCTCATCAAGCCAACCAA | + | 956 |
|  | 3 MKWV R1 | 2636 | TTGTTATGACCTTAGACCTCG | - |  |
|  | 3 MKWV R2 | 2607 | CGTCCTTTATTCTCTGAGTGC | - |  |
|  | 4 MKWV F1 | 2522 | ACATTCTGTCAAGATTTTGCCAC | + | 934 |
|  | 4 MKWV R1 | 3485 | GCAGCCTCTTGATTCTAAAGC | - |  |
|  | 4 MKWV R2 | 3456 | CTGACAACTTTAGCCCTTGCC | - |  |
|  | 5 MKWV F1 | 3264 | GCAGGGAATCCTCCATAACAC | + | 887 |
|  | 5 MKWV F2 | 3288 | CTCACTGCTGCACACTGTC | + |  |
|  | 5 MKWV R | 4175 | TTAATGCCTCTTCACCGGACT | - |  |
|  | 6 MKWV F1 | 4015 | GAGAGCTGCACAGTATCCCC | + | 525 |
|  | 6 MKWV R1 | 4968 | CTGCTCCTTTGTCGAGTCC | - |  |
|  | 6 MKWV R2 | 4540 | GAATCTCCCAGAACTTGCAC | - |  |
|  | 7 MKWV F1 | 4459 | AAGTTGGATGAGATAGCCTT | + | 880 |
|  | 7 MKWV R1 | 5361 | CACATCGCAAGAGTTCAGC | - |  |
|  | 7 MKWV R2 | 5339 | TCCCATGTCTGAGCACCAAG | - |  |
|  | 8 MKWV F1 | 5279 | TGGTCGCAGTAACAGTCTCA | + | 1009 |
|  | 8 MKWV R1 | 6346 | GACAAGGGCCAACCATCACA | - |  |
|  | 8 MKWV R2 | 6288 | TCTAGCCGAGCATGTCATCC | - |  |
|  | 9 MKWV F1 | 6075 | CAATCTCAGCCTGATGTCC | + | 363 |
|  | 9 MKWV F2 | 6049 | GGACCCAGCTACTACAAGGA | + |  |
|  | 9 MKWV R2 | 6412 | AAAGTCCGCCCATTACCAA | - |  |
| M segment | 10 MKWV F1 | 1 | TACACAAAGAACTCGCTTCA | + | 893 |
|  | 10 MKWV R1 | 977 | CTGCGAATAGCACGATCCTT | - |  |
|  | 10 MKWV R2 | 894 | CCTTCCTGCTGCATTCACTC | - |  |
|  | 11 MKWV F1 | 727 | TGAGTTTGTTTGCACCGACCA | + | 880 |
|  | 11 MKWV F2 | 833 | ACCTGTAAAGCTATGACTGCCAA | + |  |
|  | 11 MKWV R2 | 1713 | CCCCTTCACTGAGCAAACCA | - |  |
|  | 12 MKWV F1 | 1673 | TCCACTGACCAGATCACTTGCG | + | 1024 |
|  | 12 MKWV R1 | 2751 | GCACCATAGTCCCCGTAGCTC | - |  |
|  | 12 MKWV R2 | 2697 | AGTCCCTGTGTTGCACGAAT | - |  |
|  | 13 MKWV F1 | 2369 | GAGATCAGGTGCCCATCACA | + | 942 |
|  | 13 MKWV R1 | 2590 | CAAGCCTATCAAGGTGATCCTC | + |  |
|  | 13 MKWV R2 | 3311 | TACACAAAGAAACGGCGCAA | - |  |
| S segment | 14 MKWV F | 1 | GCCCCTCTTTGGAGTTCTGT | + | 686 |
|  | 14 MKWV R1 | 905 | CACTTTGACATGTTCCCCGAT | - |  |
|  | 14 MKWV R2 | 687 | TCAACTAGGCCAAGGTCCATC | - |  |
|  | 15 MKWV F | 461 | CCATGATGCACCCAAGCTTCG | + | 882 |
|  | 15 MKWV R1 | 1414 | TTAGAGAGTTCTGCCGCTCCA | - |  |
|  | 15 MKWV R2 | 1343 | CTCATATTCAAGGCCGCCAAG | - |  |
|  | 16 MKWV F2 | 900 | ATTAAATCGGGGAACATGTCA | - | 978 |
|  | 16 MKWV F1 | 997 | CCTTCTTTCCTTTCGGCTT | + |  |
|  | 16 MKWV R | 1878 | TTTAAAGACCCCCTTTCATTG |  |  |
| Inflammatory cytokine assay |  |  |  |  |  |
| IL-1β | F |  | CCAAACCTCTTCGAGGCACA | + | 85 |
|  | R |  | GCTGCTTCAGACACTTGAGC | - |  |
| IL-6 | F |  | AGAGGCACTGGCAGAAAACA | + | 95 |
|  | R |  | TCACCAGGCAAGTCTCCTCA | - |  |
| IL-8 | F |  | GAAGTTTTTGAAGAGGGCTGAGA | + | 90 |
|  | R |  | TGCTTGAAGTTTCACTGGCATC | - |  |
| TNFα | F |  | GCCCATGTTGTAGCAAACCC | + | 133 |
|  | R |  | TGAGGTACAGGCCCTCTGAT | - |  |
| Actin beta | F |  | GAGACCGCGTCCGCC | + | 87 |
|  | R |  | ATCATCCATGGTGAGCTGGC | - |  |

**Table S2. Reference sequences used in the present study.**

| Genus | Virus | Strain | GenBank accession No. |
| --- | --- | --- | --- |
| *Phlebovirus* |  |  |  |
|  | Mukawa virus | HLJ1 | PQ215521- PQ215523 |
|  | Mukawa virus | NE-FZ2 | ON408120-ON408122 |
|  | Mukawa virus | NE-FZ3 | ON408123-ON408125 |
|  | Mukawa virus | NE-ShL2 | ON408126-ON408128 |
|  | Mukawa virus | GH1 | OR730564, OR730567, OR730570 |
|  | Mukawa virus | NE-YC4 | ON408117-ON408119 |
|  | Mukawa virus | XQ1 | OR730563, OR730566, OR730569 |
|  | Mukawa virus | YKS1 | OR730562, OR730565, OR730568 |
|  | Mukawa virus | NE-TH3 | ON408114-ON408116 |
|  | Mukawa virus | 346-6443 | OM066888-OM066890 |
|  | Mukawa virus | NE-DH3 | ON408129-ON408131 |
|  | Mukawa virus | — | MZ532499-MZ532501 |
|  | Mukawa virus | MKW73 | NC043509-NC043511 |
|  | Kuriyama virus | CZCT80Q | LC133178-LC133180 |
|  | Mudanjiang phlebovirus | NE-FZ3 | ON408132-ON408134 |
|  | Mudanjiang phlebovirus | NE-MDJ2 | ON408135-ON408137 |
|  | Qingdao tick phlebovirus | SDQDR01 | OQ513651-OQ513653 |
|  | Alxa tick phlebovirus | ZQ16-15 | OP313002-OP313004 |
|  | Sand fever Naples-like virus | — | HM566182-HM566184 |
|  | Itaporanga virus | original | NC055321-NC055323 |
|  | Adana virus | 195 | NC029127-NC029127 |
|  | Cacao virus | VP 437R | MK330757, MK896596, MK896595 |
|  | Rift Valley fever virus | Lunyo | KU167025-KU167027 |
| *Bandavirus* |  |  |  |
|  | Razdan virus | LEIV-Arm2741 | KC335496-KC335498 |
|  | Bhanja virus | ibAr2709 | JX961616-JX961618 |
|  | Kismayo virus | LEIV3641A | KM114252-KM114254 |
|  | Lone Star virus | TMA 1381 | KC589005-KC589007 |
|  | Hunter Island virus | — | KF848980-KF848982 |
|  | Heartland virus | TN | KJ740146-KJ740148 |
|  | Guertu virus | DXM | KT328591-KT328593 |
|  | SFTSV |  | HM745930- HM745932 |
| *Uukuvirus* |  |  |  |
|  | Kaisodi virus | G14132 | MG581739-MG581739 |
|  | Tacheng Tick Virus 2 | TC252 | KM817684, KM817744 |
|  | American dog tick phlebovirus | F16 | KM048311-KM048311 |
|  | Lihan Tick Virus | LH-1 | KM817672, KM817736 |
|  | Yongjia Tick Virus | YJ1-1 | KM817704, KM817764 |
|  | Dabieshan tick virus | D3 | KM817666, KM817733 |
|  | Huangpi Tick Virus | H114-17 | KM817668, KM817707, KM817735 |
|  | Silverwater virus | Can131 | KM114255-KM114257 |
|  | Kabuto mountain virus | T32 | LC153711-LC153713 |
|  | Rukutama virus | LEIV-6269C | KF892052-KF892054 |
|  | Grand Arbaud virus | Argas 27 | JF838327-JF838327 |
|  | Precarious point virus | — | HM566179- HM566181 |
|  | Murre virus | Murre H | JF838330-JF838332 |
|  | EgAN 1825-61 virus | — | HM566158-HM566160 |
|  | Zaliv Terpenia virus | — | HM566191-HM566193 |
|  | Uukuniemi virus | Potepli 63 | KM114246-KM114248 |
| *Ixovirus* |  |  |  |
|  | Fairhair virus | NOR/A2/Bronnoya/2014 | MF141052-MF141053 |
|  | Blacklegged tick phlebovirus-1 | H12 | KM048313-KM048314 |
|  | Blacklegged tick phlebovirus 3 | A1 | KU230449-KU230450 |

**Table S3. Prevalence of Mukawa virus strain HLJ1 in *Ixodes persulcatus* ticks.**

| Tick sample | Number of ticks | Tick pools  (No. positive/Total) | Engorgement status | Prevalence (%)^*^ | 95% CI |
| --- | --- | --- | --- | --- | --- |
| Female | 164 | 3/17 | — | 1.87 | (0.50-5.05) |
| Male | 279 | 5/28 | — | 1.91 | (0.72-4.22) |
| Total | 443 | 8/45 | — | 1.92 | (0.90-3.63) |

**Table S4. Nucleotide (upper right) and amino acid (lower left) sequence identity of Mukawa virus RNA-dependent RNA polymerase calculated using the Sequence Identity Matrix programme in Bioedit (v7.0.5.3).**

| Virus | 1 | 2 | 3 | 4 | 5 | 6 | 7 | 8 | 9 | 10 | 11 | 12 | 13 | 14 | 15 | 16 | 17 | 18 |
| --- | --- | --- | --- | --- | --- | --- | --- | --- | --- | --- | --- | --- | --- | --- | --- | --- | --- | --- |
| 1. PQ215521 Mukawa virus HLJ1 |  | 95.9 | 95.4 | 95.3 | 95.5 | 96.0 | 99.8 | 99.8 | 98.1 | 95.1 | 93.0 | 95.1 | 92.2 | 82.3 | 75.8 | 75.8 | 74.0 | 75.9 |
| 2. OR730564 Mukawa virus GH1 | 99.7 |  | 95.8 | 95.9 | 96.0 | 97.2 | 96.1 | 96.1 | 96.3 | 94.5 | 93.0 | 95.6 | 92.5 | 83.0 | 76.3 | 76.3 | 74.1 | 76.4 |
| 3. OR730562 Mukawa virus YKS1 | 99.3 | 99.3 |  | 96.3 | 98.7 | 95.7 | 95.6 | 95.6 | 95.7 | 94.3 | 93.0 | 98.2 | 93.0 | 83.1 | 75.8 | 76.0 | 74.0 | 75.9 |
| 4. OR730563 Mukawa virus XQ1 | 99.1 | 99.2 | 99.2 |  | 96.3 | 95.6 | 95.5 | 95.5 | 95.5 | 94.1 | 93.1 | 96.0 | 92.8 | 82.8 | 75.8 | 76.0 | 74.1 | 76.0 |
| 5. ON408114 Mukawa virus NE-TH3 | 99.2 | 99.3 | 99.6 | 99.2 |  | 95.7 | 95.7 | 95.7 | 95.7 | 94.2 | 93.1 | 98.9 | 93.0 | 83.1 | 75.9 | 75.8 | 74.0 | 75.9 |
| 6. ON408117 Mukawa virus NE-YC4 | 99.6 | 99.7 | 99.2 | 99.2 | 99.2 |  | 96.2 | 96.2 | 96.3 | 94.3 | 92.6 | 95.4 | 92.4 | 82.6 | 75.9 | 76.0 | 73.8 | 76.0 |
| 7. ON408120 Mukawa virus N3-FZ2 | 100.0 | 99.7 | 99.3 | 99.1 | 99.2 | 99.6 |  | 100.0 | 98.3 | 95.3 | 93.2 | 95.2 | 92.4 | 82.5 | 76.0 | 76.0 | 74.1 | 76.1 |
| 8. ON408123 Mukawa virus NE-FZ3 | 100.0 | 99.7 | 99.3 | 99.1 | 99.2 | 99.6 | 100.0 |  | 98.3 | 95.3 | 93.2 | 95.2 | 92.4 | 82.5 | 76.0 | 76.0 | 74.1 | 76.1 |
| 9. ON408126 Mukawa virus NE-ShL2 | 99.7 | 99.6 | 99.2 | 99.0 | 99.1 | 99.5 | 99.7 | 99.7 |  | 95.7 | 93.2 | 95.4 | 92.4 | 82.6 | 75.7 | 75.9 | 74.0 | 75.9 |
| 10. ON408129 Mukawa virusNE-DH3 | 99.3 | 99.2 | 98.9 | 98.8 | 98.8 | 99.1 | 99.3 | 99.3 | 99.4 |  | 95.5 | 94.4 | 92.9 | 82.7 | 76.1 | 76.1 | 74.0 | 76.2 |
| 11. MZ532501 Mukawa virus | 98.7 | 98.8 | 98.7 | 98.6 | 98.7 | 98.7 | 98.7 | 98.7 | 98.6 | 98.8 |  | 93.3 | 93.1 | 82.9 | 76.3 | 76.2 | 73.9 | 76.4 |
| 12. OM066888 Mukawa virus 346-6443 | 99.2 | 99.3 | 99.7 | 99.4 | 99.7 | 99.2 | 99.2 | 99.2 | 99.1 | 98.9 | 98.8 |  | 92.9 | 82.9 | 75.9 | 75.8 | 74.0 | 75.9 |
| 13. NC043510 Mukawa virus MKW73 | 99.1 | 99.2 | 99.0 | 98.9 | 99.1 | 99.1 | 99.1 | 99.1 | 99.0 | 98.9 | 99.2 | 99.1 |  | 82.9 | 76.1 | 76.2 | 73.6 | 76.3 |
| 14. LC133178 Kuriyama virus CZCT80Q | 95.2 | 95.2 | 95.3 | 95.0 | 95.2 | 95.2 | 95.2 | 95.2 | 95.2 | 95.1 | 95.0 | 95.2 | 95.3 |  | 77.3 | 77.4 | 74.0 | 77.3 |
| 15. ON408135 Mudanjiang phlebovirus NE-MDJ2 | 88.8 | 89.0 | 88.8 | 88.9 | 89.0 | 88.9 | 88.8 | 88.8 | 88.8 | 88.7 | 88.6 | 88.9 | 88.9 | 88.4 |  | 95.2 | 74.4 | 98.9 |
| 16. ON408132 Mudanjiang phlebovirus NE-FZ3 | 88.6 | 88.7 | 88.6 | 88.7 | 88.8 | 88.7 | 88.6 | 88.6 | 88.6 | 88.5 | 88.4 | 88.7 | 88.7 | 88.1 | 99.4 |  | 74.3 | 94.9 |
| 17. OP313002 Alxa tick phlebovirus | 82.7 | 82.6 | 82.8 | 82.7 | 83.0 | 82.7 | 82.7 | 82.7 | 82.7 | 82.7 | 82.5 | 83.0 | 82.9 | 82.9 | 83.9 | 83.7 |  | 74.4 |
| 18. OQ513651 Qingdao tick phlebovirus | 88.8 | 89.0 | 88.8 | 88.9 | 89.0 | 88.9 | 88.8 | 88.8 | 88.8 | 88.7 | 88.6 | 88.9 | 88.9 | 88.3 | 99.6 | 99.2 | 83.9 |  |

**Table S5.** **Nucleotide (upper right) and amino acid (lower left) sequence identity of Mukawa virus glycoprotein precursor calculated using the Sequence Identity Matrix programme in Bioedit (v7.0.5.3).**

| Virus | 1 | 2 | 3 | 4 | 5 | 6 | 7 | 8 | 9 | 10 | 11 | 12 | 13 | 14 | 15 | 16 | 17 | 18 |
| --- | --- | --- | --- | --- | --- | --- | --- | --- | --- | --- | --- | --- | --- | --- | --- | --- | --- | --- |
| 1. PQ215521 Mukawa virus HLJ1 |  | 93.5 | 91.7 | 91.0 | 91.3 | 91.6 | 99.9 | 99.9 | 96.2 | 98.2 | 97.0 | 92.2 | 92.1 | 83.9 | 68.7 | 68.6 | 63.1 | 68.5 |
| 2. OR730564 Mukawa virus GH1 | 98.5 |  | 91.9 | 91.6 | 91.1 | 91.5 | 93.5 | 93.5 | 93.6 | 93.8 | 93.2 | 91.6 | 91.6 | 84.0 | 68.2 | 68.1 | 63.1 | 68.1 |
| 3. OR730562 Mukawa virus YKS1 | 98.4 | 97.8 |  | 96.5 | 92.1 | 92.1 | 91.7 | 91.7 | 91.8 | 91.8 | 91.6 | 91.3 | 91.3 | 83.7 | 68.3 | 68.3 | 63.1 | 68.3 |
| 4. OR730563 Mukawa virus XQ1 | 97.9 | 97.5 | 98.3 |  | 91.8 | 92.2 | 91.0 | 91.0 | 91.4 | 91.2 | 91.0 | 91.1 | 91.1 | 83.6 | 68.4 | 68.3 | 63.4 | 68.3 |
| 5. ON408114 Mukawa virus NE-TH3 | 98.2 | 97.6 | 98.4 | 97.9 |  | 96.0 | 91.2 | 91.2 | 91.2 | 91.5 | 91.0 | 90.7 | 90.7 | 83.7 | 68.3 | 68.2 | 62.7 | 68.1 |
| 6. ON408117 Mukawa virus NE-YC4 | 98.1 | 97.5 | 98.1 | 97.9 | 98.9 |  | 91.6 | 91.6 | 91.5 | 91.8 | 91.2 | 90.7 | 90.7 | 83.9 | 68.4 | 68.3 | 63.1 | 68.2 |
| 7. ON408120 Mukawa virus N3-FZ2 | 100.0 | 98.5 | 98.4 | 97.9 | 98.2 | 98.1 |  | 100.0 | 96.2 | 98.3 | 97.1 | 92.2 | 92.1 | 84.0 | 68.7 | 68.6 | 63.1 | 68.5 |
| 8. ON408123 Mukawa virus NE-FZ3 | 100.0 | 98.5 | 98.4 | 97.9 | 98.2 | 98.1 | 100.0 |  | 96.2 | 98.3 | 97.1 | 92.2 | 92.1 | 84.0 | 68.7 | 68.6 | 63.1 | 68.5 |
| 9. ON408126 Mukawa virus NE-ShL2 | 98.5 | 97.9 | 97.8 | 97.5 | 97.8 | 97.7 | 98.5 | 98.5 |  | 96.7 | 96.5 | 92.0 | 92.0 | 83.4 | 68.3 | 68.1 | 62.8 | 68.2 |
| 10. ON408129 Mukawa virusNE-DH3 | 99.4 | 98.3 | 98.2 | 97.8 | 98.0 | 97.9 | 99.4 | 99.4 | 98.5 |  | 97.3 | 92.2 | 92.1 | 83.8 | 68.6 | 68.5 | 63.0 | 68.5 |
| 11. MZ532501 Mukawa virus | 99.1 | 97.9 | 97.9 | 97.5 | 97.8 | 97.5 | 99.1 | 99.1 | 97.9 | 98.7 |  | 92.2 | 92.1 | 83.7 | 68.4 | 68.3 | 63.0 | 68.2 |
| 12. OM066888 Mukawa virus 346-6443 | 98.7 | 98.0 | 98.1 | 98.2 | 97.9 | 97.9 | 98.7 | 98.7 | 98.0 | 98.5 | 98.0 |  | 99.9 | 83.1 | 68.2 | 68.2 | 63.2 | 68.3 |
| 13. NC043510 Mukawa virus MKW73 | 98.6 | 98.1 | 98.1 | 98.2 | 97.9 | 97.9 | 98.6 | 98.6 | 98.0 | 98.4 | 97.9 | 99.9 |  | 83.2 | 68.2 | 68.2 | 63.2 | 68.3 |
| 14. LC133178 Kuriyama virus CZCT80Q | 94.1 | 94.0 | 93.9 | 93.7 | 93.9 | 93.8 | 94.1 | 94.1 | 93.3 | 93.9 | 93.7 | 93.7 | 93.7 |  | 69.1 | 69.4 | 63.0 | 69.2 |
| 15. ON408135 Mudanjiang phlebovirus NE-MDJ2 | 71.3 | 70.7 | 70.6 | 70.7 | 70.6 | 70.8 | 71.3 | 71.3 | 70.7 | 70.9 | 70.9 | 70.8 | 70.7 | 70.3 |  | 98.7 | 63.6 | 98.4 |
| 16. ON408132 Mudanjiang phlebovirus NE-FZ3 | 71.2 | 70.6 | 70.6 | 70.6 | 70.6 | 70.7 | 71.2 | 71.2 | 70.6 | 70.8 | 70.8 | 70.7 | 70.6 | 70.2 | 99.2 |  | 63.7 | 98.5 |
| 17. OP313002 Alxa tick phlebovirus | 62.3 | 62.0 | 62.5 | 62.5 | 62.3 | 62.6 | 62.3 | 62.3 | 62.2 | 62.0 | 62.0 | 62.5 | 62.4 | 61.6 | 63.3 | 63.3 |  | 63.6 |
| 18. OQ513651 Qingdao tick phlebovirus | 71.0 | 70.5 | 70.4 | 70.5 | 70.4 | 70.6 | 71.0 | 71.0 | 70.5 | 70.6 | 70.6 | 70.6 | 70.5 | 70.1 | 99.4 | 99.2 | 63.2 |  |

**Table S6.** **Nucleotide (upper right) and amino acid (lower left) sequence identity of Mukawa virus nucleoprotein calculated using the Sequence Identity Matrix programme in Bioedit (v7.0.5.3).**

| Virus | 1 | 2 | 3 | 4 | 5 | 6 | 7 | 8 | 9 | 10 | 11 | 12 | 13 | 14 | 15 | 16 | 17 | 18 |
| --- | --- | --- | --- | --- | --- | --- | --- | --- | --- | --- | --- | --- | --- | --- | --- | --- | --- | --- |
| 1. PQ215521 Mukawa virus HLJ1 |  | 97.4 | 97.7 | 97.5 | 97.9 | 97.0 | 100.0 | 100.0 | 97.7 | 97.9 | 96.7 | 97.8 | 96.2 | 87.6 | 78.6 | 79.0 | 72.5 | 79.3 |
| 2. OR730564 Mukawa virus GH1 | 100.0 |  | 97.8 | 98.5 | 98.1 | 98.2 | 97.4 | 97.4 | 97.8 | 97.1 | 96.6 | 97.9 | 96.1 | 88.1 | 78.4 | 79.1 | 73.1 | 79.4 |
| 3. OR730562 Mukawa virus YKS1 | 99.5 | 99.5 |  | 98.2 | 99.1 | 97.4 | 97.7 | 97.7 | 98.3 | 97.7 | 96.5 | 99.0 | 96.6 | 87.5 | 79.0 | 79.4 | 72.5 | 79.7 |
| 4. OR730563 Mukawa virus XQ1 | 100.0 | 100.0 | 99.5 |  | 98.5 | 99.1 | 97.5 | 97.5 | 97.9 | 97.3 | 96.2 | 98.3 | 96.5 | 87.3 | 78.8 | 79.5 | 72.9 | 79.8 |
| 5. ON408114 Mukawa virus NE-TH3 | 100.0 | 100.0 | 99.5 | 100.0 |  | 97.7 | 97.9 | 97.9 | 98.3 | 97.7 | 96.5 | 99.5 | 96.6 | 87.5 | 79.0 | 79.4 | 72.7 | 79.7 |
| 6. ON408117 Mukawa virus NE-YC4 | 100.0 | 100.0 | 99.5 | 100.0 | 100.0 |  | 97.0 | 97.0 | 97.4 | 96.7 | 95.6 | 97.5 | 95.9 | 87.6 | 78.7 | 79.7 | 72.9 | 79.9 |
| 7. ON408120 Mukawa virus N3-FZ2 | 100.0 | 100.0 | 99.5 | 100.0 | 100.0 | 100.0 |  | 100.0 | 97.7 | 97.9 | 96.7 | 97.8 | 96.2 | 87.6 | 78.6 | 79.0 | 72.5 | 79.3 |
| 8. ON408123 Mukawa virus NE-FZ3 | 100.0 | 100.0 | 99.5 | 100.0 | 100.0 | 100.0 | 100.0 |  | 97.7 | 97.9 | 96.7 | 97.8 | 96.2 | 87.6 | 78.6 | 79.0 | 72.5 | 79.3 |
| 9. ON408126 Mukawa virus NE-ShL2 | 100.0 | 100.0 | 99.5 | 100.0 | 100.0 | 100.0 | 100.0 | 100.0 |  | 97.9 | 96.7 | 98.2 | 96.9 | 87.7 | 78.8 | 79.3 | 72.7 | 79.5 |
| 10. ON408129 Mukawa virusNE-DH3 | 100.0 | 100.0 | 99.5 | 100.0 | 100.0 | 100.0 | 100.0 | 100.0 | 100.0 |  | 98.2 | 97.5 | 96.7 | 87.3 | 79.0 | 79.4 | 72.7 | 79.7 |
| 11. MZ532501 Mukawa virus | 100.0 | 100.0 | 99.5 | 100.0 | 100.0 | 100.0 | 100.0 | 100.0 | 100.0 | 100.0 |  | 96.3 | 96.3 | 88.0 | 78.4 | 78.6 | 73.2 | 78.8 |
| 12. OM066888 Mukawa virus 346-6443 | 100.0 | 100.0 | 99.5 | 100.0 | 100.0 | 100.0 | 100.0 | 100.0 | 100.0 | 100.0 | 100.0 |  | 96.5 | 87.3 | 79.0 | 79.4 | 72.8 | 79.7 |
| 13. NC043510 Mukawa virus MKW73 | 99.5 | 99.5 | 99.1 | 99.5 | 99.5 | 99.5 | 99.5 | 99.5 | 99.5 | 99.5 | 99.5 | 99.5 |  | 87.2 | 78.0 | 78.4 | 72.3 | 78.7 |
| 14. LC133178 Kuriyama virus CZCT80Q | 93.1 | 93.1 | 92.7 | 93.1 | 93.1 | 93.1 | 93.1 | 93.1 | 93.1 | 93.1 | 93.1 | 93.1 | 92.7 |  | 77.9 | 77.8 | 71.2 | 77.8 |
| 15. ON408135 Mudanjiang phlebovirus NE-MDJ2 | 84.6 | 84.6 | 84.2 | 84.6 | 84.6 | 84.6 | 84.6 | 84.6 | 84.6 | 84.6 | 84.6 | 84.6 | 84.2 | 81.3 |  | 97.1 | 73.2 | 96.9 |
| 16. ON408132 Mudanjiang phlebovirus NE-FZ3 | 84.2 | 84.2 | 84.6 | 84.2 | 84.2 | 84.2 | 84.2 | 84.2 | 84.2 | 84.2 | 84.2 | 84.2 | 83.8 | 80.9 | 99.1 |  | 72.7 | 99.1 |
| 17. OP313002 Alxa tick phlebovirus | 75.7 | 75.7 | 75.7 | 75.7 | 75.7 | 75.7 | 75.7 | 75.7 | 75.7 | 75.7 | 75.7 | 75.7 | 75.7 | 74.4 | 75.3 | 75.3 |  | 73.1 |
| 18. OQ513651 Qingdao tick phlebovirus | 84.2 | 84.2 | 84.6 | 84.2 | 84.2 | 84.2 | 84.2 | 84.2 | 84.2 | 84.2 | 84.2 | 84.2 | 83.8 | 80.9 | 99.5 | 99.5 | 75.3 |  |

**Table S7. Nucleotide (upper right) and amino acid (lower left) sequence identity of Mukawa virus non-structural proteins calculated using the Sequence Identity Matrix programme in Bioedit (v7.0.5.3).**

| Virus | 1 | 2 | 3 | 4 | 5 | 6 | 7 | 8 | 9 | 10 | 11 | 12 | 13 | 14 | 15 | 16 | 17 | 18 |
| --- | --- | --- | --- | --- | --- | --- | --- | --- | --- | --- | --- | --- | --- | --- | --- | --- | --- | --- |
| 1. PQ215521 Mukawa virus HLJ1 |  | 93.7 | 91.5 | 92.1 | 92.6 | 94.7 | 99.7 | 99.9 | 92.1 | 92.7 | 88.5 | 91.7 | 91.2 | 74.4 | 61.4 | 61.4 | 61.0 | 54.5 |
| 2. OR730564 Mukawa virus GH1 | 94.7 |  | 91.9 | 91.9 | 92.8 | 97.6 | 93.4 | 93.6 | 92.1 | 94.7 | 89.9 | 91.7 | 91.3 | 74.1 | 60.8 | 60.6 | 55.5 | 60.2 |
| 3. OR730562 Mukawa virus YKS1 | 92.6 | 92.6 |  | 94.1 | 95.1 | 91.9 | 91.3 | 91.4 | 93.0 | 92.5 | 92.3 | 95.4 | 90.9 | 71.8 | 61.3 | 61.2 | 54.6 | 61.1 |
| 4. OR730563 Mukawa virus XQ1 | 94.7 | 94.1 | 94.4 |  | 96.4 | 92.0 | 91.8 | 92.0 | 94.6 | 91.8 | 90.7 | 93.2 | 91.7 | 73.4 | 61.0 | 60.9 | 54.2 | 60.8 |
| 5. ON408114 Mukawa virus NE-TH3 | 95.2 | 94.7 | 95.0 | 98.2 |  | 92.7 | 92.3 | 92.5 | 95.1 | 92.2 | 90.3 | 93.6 | 91.3 | 73.9 | 60.9 | 60.9 | 54.5 | 60.8 |
| 6. ON408117 Mukawa virus NE-YC4 | 95.8 | 98.2 | 93.2 | 95.2 | 95.2 |  | 94.4 | 94.6 | 92.5 | 96.4 | 90.6 | 92.1 | 91.4 | 74.0 | 61.4 | 61.4 | 55.2 | 61.0 |
| 7. ON408120 Mukawa virus N3-FZ2 | 99.4 | 94.1 | 92.0 | 94.1 | 94.7 | 95.2 |  | 99.8 | 91.8 | 92.4 | 90.2 | 91.4 | 91.1 | 74.2 | 61.4 | 61.4 | 54.5 | 61.0 |
| 8. ON408123 Mukawa virus NE-FZ3 | 100.0 | 94.7 | 92.6 | 94.7 | 95.2 | 95.8 | 99.4 |  | 92.0 | 92.6 | 90.2 | 91.6 | 91.1 | 74.3 | 61.4 | 61.4 | 54.6 | 61.0 |
| 9. ON408126 Mukawa virus NE-ShL2 | 95.5 | 95.0 | 94.4 | 96.4 | 96.7 | 95.8 | 95.2 | 95.5 |  | 92.7 | 90.7 | 92.4 | 90.9 | 73.9 | 60.6 | 60.7 | 53.7 | 60.5 |
| 10. ON408129 Mukawa virusNE-DH3 | 95.8 | 95.8 | 94.4 | 95.8 | 95.8 | 97.6 | 95.2 | 95.8 | 96.7 |  | 92.5 | 93.7 | 91.6 | 73.4 | 60.7 | 60.2 | 54.6 | 60.1 |
| 11. MZ532501 Mukawa virus | 90.3 | 89.1 | 92.0 | 90.0 | 89.7 | 89.7 | 88.8 | 88.5 | 90.0 | 90.8 |  | 96.0 | 92.5 | 72.6 | 61.2 | 60.8 | 53.6 | 60.9 |
| 12. OM066888 Mukawa virus 346-6443 | 91.4 | 91.7 | 95.8 | 93.8 | 94.4 | 92.0 | 91.1 | 91.4 | 93.2 | 93.2 | 94.7 |  | 92.0 | 72.1 | 61.2 | 60.7 | 54.0 | 60.8 |
| 13. NC043510 Mukawa virus MKW73 | 93.5 | 93.8 | 92.3 | 93.5 | 93.5 | 94.1 | 93.5 | 93.5 | 93.8 | 94.7 | 91.7 | 92.3 |  | 74.4 | 61.4 | 61.5 | 55.6 | 61.4 |
| 14. LC133178 Kuriyama virus CZCT80Q | 73.2 | 74.1 | 70.8 | 72.6 | 73.5 | 73.8 | 73.2 | 73.2 | 73.8 | 73.8 | 71.7 | 70.5 | 75.0 |  | 62.5 | 61.9 | 54.9 | 61.8 |
| 15. ON408135 Mudanjiang phlebovirus NE-MDJ2 | 55.8 | 55.8 | 57.3 | 56.1 | 56.7 | 56.4 | 55.8 | 55.8 | 57.0 | 56.4 | 57.3 | 57.0 | 56.1 | 56.1 |  | 95.2 | 57.2 | 95.5 |
| 16. ON408132 Mudanjiang phlebovirus NE-FZ3 | 56.7 | 56.1 | 57.6 | 57.0 | 57.6 | 56.7 | 56.7 | 56.7 | 57.3 | 56.7 | 57.3 | 56.7 | 56.4 | 55.5 | 97.0 |  | 58.1 | 98.9 |
| 17. OP313002 Alxa tick phlebovirus | 56.7 | 48.6 | 50.1 | 49.8 | 50.1 | 48.9 | 50.4 | 50.4 | 49.8 | 49.5 | 48.9 | 49.5 | 48.9 | 48.3 | 52.6 | 53.8 |  | 57.8 |
| 18. OQ513651 Qingdao tick phlebovirus | 50.0 | 56.1 | 57.6 | 57.0 | 57.6 | 56.7 | 56.7 | 56.7 | 57.3 | 56.7 | 57.9 | 57.3 | 56.4 | 55.5 | 97.9 | 98.5 | 53.2 |  |

**Table S8.** **The DNA sequences employed for modifying membranes in the present study.**

| Name | Sequence (5' to 3') |
| --- | --- |
| chol-11A | chol-TTTTTTTTTTTTTTTTTTTTTTTTTTTTTTGCTACAGGCTTGACGGGGAAAG  CCCAAAGGGC |
| chol-41A | chol-TTTTTTTTTTTTTTTTTTTTTTTTTTTTTTAAGGAGCGTAGGAGCACTAACA  ACGCCAGCAG |
| chol-69A | chol-TTTTTTTTTTTTTTTTTTTTTTTTTTTTTTCAACGCTCAACGCGAGAAAACT  TTCGCTGAGA |
| chol-101A | chol-TTTTTTTTTTTTTTTTTTTTTTTTTTTTTTCGGAATAACAATGAAATAGCAAT  AAAACAGGG |
| chol-128A | chol-TTTTTTTTTTTTTTTTTTTTTTTTTTTTTTAACACTGAATAGGTGTATCACCG  TTCATTAAA |
| chol-158A | chol-TTTTTTTTTTTTTTTTTTTTTTTTTTTTTTATGGTTTAAGGACAGATGAACGG  TAGTACAAC |
| chol-186A | chol-TTTTTTTTTTTTTTTTTTTTTTTTTTTTTTTATATTTTTTGCGGATGGCTTAGA  AGGAAGCC |
| chol-218A | chol-TTTTTTTTTTTTTTTTTTTTTTTTTTTTTTAAAACGACGTATCGGCCTCAGG  AATGAGCGAG |

**Table S9.** **The sequences of DNA used for intercellular connections in the present study.**

| Name | Sequence (5' to 3') |
| --- | --- |
| 22B | AGTAGAAGAACTCAAAGTGGCACATTTTTTTTTTGTCAGTCAGTCAGT  CAGTCA |
| 27B | GTCAGTCAGTCAGTCAGTCATTTTTTTTTTAGGGACATAAATCTAAAG  CATCACTTATCTAA |
| 86B | GTCAGTCAGTCAGTCAGTCATTTTTTTTTTGGAGGTTTTAGACGGGAG  AATTAATAATAAGA |
| 88B | GTCAGTCAGTCAGTCAGTCATTTTTTTTTTAATCTTACCAACGCTAACG  AGCGTTGTCTTTC |
| 139B | TCCAAAAGGAGCCTTTGAGGCTTTTTTTTTTTTTGTCAGTCAGTCAGT  CAGTCA |
| 144B | GTCAGTCAGTCAGTCAGTCATTTTTTTTTTGATCGTCAGTATCATCGCC  TGATATGACCAAC |
| 203B | GTCAGTCAGTCAGTCAGTCATTTTTTTTTTACTAGCATCGTCGGATTCT  CCGTGTGAGGGGA |
| 205B | GTCAGTCAGTCAGTCAGTCATTTTTTTTTTTATAAGCAAATATTTAAAT  TGTAATAAAGATT |
| com-22B | AGTAGAAGAACTCAAAGTGGCACATTTTTTTTTTTGACTGACTGACTG  ACTGAC |
| com-27B | TGACTGACTGACTGACTGACTTTTTTTTTTAGGGACATAAATCTAAAG  CATCACTTATCTAA |
| com-86B | TGACTGACTGACTGACTGACTTTTTTTTTTGGAGGTTTTAGACGGGAG  AATTAATAATAAGA |
| com-88B | TGACTGACTGACTGACTGACTTTTTTTTTTAATCTTACCAACGCTAACG  AGCGTTGTCTTTC |
| com-139B | TCCAAAAGGAGCCTTTGAGGCTTTTTTTTTTTTTTGACTGACTGACTG  ACTGAC |
| com-144B | TGACTGACTGACTGACTGACTTTTTTTTTTGATCGTCAGTATCATCGCC  TGATATGACCAAC |
| com-203B | TGACTGACTGACTGACTGACTTTTTTTTTTACTAGCATCGTCGGATTCT  CCGTGTGAGGGGA |
| com-205B | TGACTGACTGACTGACTGACTTTTTTTTTTTATAAGCAAATATTTAAAT  TGTAATAAAGATT |
| AF488-DNA | AF488-TTTTTTTTTTTGACTGACTGACTGACTGAC |

**Table 10.** **The DNA sequences utilized for DNA origami interconnection in the present study.**

| Name | Sequence (5' to 3') |
| --- | --- |
| 1 | CGTGAACCATCACCCAAATCAAGTGCCGTAAA |
| 2 | GAAAAACCGTCTATCAGGGCGATGAGCCCCCG |
| 3 | ATTAAAGAACGTGGACTCCAACGTGGCGAACG |
| 4 | GTTCCAGTTTGGAACAAGCGAAAGGA |
| 5 | GTCGAGGTTTTTTGGG |
| 6 | GCACTAAATAACGTGCTTTCCTCGAGCTAAAC |
| 7 | ATTTAGAGGCGCGTACTATGGTTGGACAGGAA |
| 8 | TGGCGAGACACACCCGCCGCGCTTGTTTTTAT |
| 9 | GCGGGCGCTAGGGTTTTAGTCGGGAAA |
| 10 | AGCACGTATCGGAACCCTAAAGGGGCCCACTA |
| 11 | GCTACAGGCTTGACGGGGAAAGCCCAAAGGGC |
| 12 | GTAACCACAAGGAAGGGAAGAAAGAGTCCACT |
| 13 | AGCGGTCAGAGTCTGTCCATCACG |
| 14 | AGAGCGGGTTAGAATC |
| 15 | AGGAGGCCATTTTGACGCTCAATCTTACATTG |
| 16 | CGGTACGCGCAACAGGAAAAACGCGTAATAAA |
| 17 | AATCAGTGTAATATCCAGAACAATAGAACCCT |
| 18 | GTAGCAATACTTCTTTTTATTACGCCAGTTGCGTTGCGCTCACT |
| 19 | ATACCTACGATTAAAGGGATTTTACTTTGACG |
| 20 | CAGCCATTCAGAATCCTGAGAAGTAATGCGCC |
| 21 | CTTGCTGGAGGCCACCGAGTAAAACGCTGCGC |
| 22 | AGTAGAAGAACTCAAAGTGGCACAGACAATAT |
| 23 | TAACATCACTTGCCTGCAAATTAACCGTTTTGATTAGTAAGTCTTTAATGCTTTGATCGGTGCGGGCCTCAACTGTTG |
| 24 | GGAAGGGC |
| 25 | TGGATTATGTCTGAAA |
| 26 | GCAGATTCTATCAAACCCTCAATCGCAAATCA |
| 27 | AGGGACATAAATCTAAAGCATCACTTATCTAA |
| 28 | TCTGACCTCAGTGCCACGCTGAGATAATAGAT |
| 29 | GCGAACCGAA |
| 30 | GTCAGTTGAATATCTG |
| 31 | ACCTCAAAACCAGTCACACGACCATCATGGAA |
| 32 | CAAATGAATCTGGCCAACAGAGATATTACCGC |
| 33 | GCCTGCAAGAAAGCGTAAGAATACCTATCGGC |
| 34 | GGTGAGGCGGTCAGTATTGAGGATTTAGAAGT |
| 35 | CAGAAGATAAAACAGATTTTGAATGGCTATTAATCGCCATTAAAAATACTGATAGCCCTAAAACTTTTGTTAAATCAGCTA |
| 36 | AAATTCGCATTAAAT |
| 37 | ACAGTTGATGGCAATTCATCAATAGATTATAC |
| 38 | AATATCTTGAATTATCATCATATTCCTACCAT |
| 39 | TAGAGCCGTTTGCGGAACAAAGAAAGAAATAA |
| 40 | TCAGATGAAAGGAATTGAGGAAGGCTTGCTGA |
| 41 | AAGGAGCGTAGGAGCACTAACAACGCCAGCAG |
| 42 | ATTATCATTCAATAGATAATACATTTAACACC |
| 43 | ATTAATTTTAAAAGTTTAACGTCA |
| 44 | TTGCCCGAACGTTATTAGACTTTACAAAC |
| 45 | ATTGTTTGTAATCCTG |
| 46 | TTCTGAATACCTGAGCAAAAGAAGCAAGAAAA |
| 47 | ATCAAAATAATCGCGCAGAGGCGATCATTTGA |
| 48 | AGAAATTGTTCGCCTGATTGCTTTACATAAAT |
| 49 | GATGAATATACAGTTTTTTAAATAAGGCGT |
| 50 | TTTCAATTAATGGAAGGGTTAGAACCTGATTA |
| 51 | AGTTACAATATTTGCACGTAAAACACCACCAG |
| 52 | ATAACGGACGTAGATTTTCAGGTTTGAGTAAC |
| 53 | TACATCGGGCTTCTGTAAATCGTCGCTATT |
| 54 | ACAAACATATGATGAA |
| 55 | CAAAATTAATTACATTTAACAATTATTATTCA |
| 56 | ATTACCTTTTTTAATGGAAACAGTGAATACCA |
| 57 | CAATATATGTGAGTGAATAACCTTGAGAAACA |
| 58 | TCTGAGAGACTACCTTTTTAACCTTATATAAC |
| 59 | AGAGTCAATAGTGAATTTATCAAACAATCGCA |
| 60 | ACATAGCGATAGCTTAGATTAAGATTCAAATA |
| 61 | TTAATTTTCCCTTAGAATACCTAAAT |
| 62 | GGTTGGGTCCGGCTTA |
| 63 | TATATGTATAACAACGCCAACATGATTTTCGA |
| 64 | AGACAAAGAACAGTAGGGCTTAATACCGACAA |
| 65 | TATTTTAGTACAAATTCTTACCAGGACGACAA |
| 66 | TTAATGGTTAGAAAAAGCCTGTTTGAACGCGC |
| 67 | ACCGTGTGATTTTTTAACAGTACCTTT |
| 68 | GCCATATTAATGCTGATGCAAATCATCATAGG |
| 69 | CAACGCTCAACGCGAGAAAACTTTCGCTGAGA |
| 70 | ATGCGTTATTAATTTCATCTTCTGCCTTGAAA |
| 71 | ATAATTACTTGAAATACCG |
| 72 | TAAATAAGAATAAACACTGAACAAGAATTTTCGTATTAAATCCT |
| 73 | GCAGAGGCTAATTTAG |
| 74 | GCCAGTAAGCAAATCAGATATAGACTAAGAAC |
| 75 | AAGGTAAAATCGTAGGAATCATTAGACTTGCG |
| 76 | TAAACAACTCATCGAGAACAAGCAGATTAGTT |
| 77 | CTGTTTATTCCAAGAACGGGTATTTTATCCTG |
| 78 | AAATACCAATCAATAATCGGC |
| 79 | AATAGCAATAAGAGAATATAAAGTTGAGAATC |
| 80 | TTATTTTCGTAATTCTGTCCAGACTATAAAGC |
| 81 | TACCGCACATGTTCAGCTAATGCAAGTATCAT |
| 82 | CTTATCATCAACAATAGATAAGTCCCGGAATC |
| 83 | TACGAGCATGTAGAAAATATCCCATCCTAATTTTTCGAACCACCAGAATTCGACAAC |
| 84 | CCGGTATTAGGCTTAT |
| 85 | GCGAGGCGAAGTCAGAGGGTAATTGAGATAAC |
| 86 | GGAGGTTTTAGACGGGAGAATTAATAATAAGA |
| 87 | GCTATTTTAGAGAGAATAACATAAGCTATCTT |
| 88 | AATCTTACCAACGCTAACGAGCGTTGTCTTTC |
| 89 | CTTTCCAGAGCCTAATTATCCCAATCCAAATA |
| 90 | CCTGAACATTTTAGCGAACCTCCCCCGCGCCC |
| 91 | AAGCGCATTGAAGCCTTAAATCAAAGCCGTTT |
| 92 | GCCTTTACGCACCCAGCTACAATTAAACCAAG |
| 93 | TTTTTTGTTTAACGTCAAAAATGAAAGCAGAT |
| 94 | ATATCAGAGAGCGCTA |
| 95 | CCACAAGAATGGTTTACCAGCGCCCATTCAAC |
| 96 | GCAAGAAAGTTTATTTTGTCACAATTGACGGA |
| 97 | ACCGAAGCTATAAAAGAAACGCAACACCGTCA |
| 98 | AGCCGAACACGTAGAAAATACATAGAGCCAGC |
| 99 | AACCGAGGAAACGCAATTAAG |
| 100 | AAATTCATATTGAGTTAAGCCCAACTGAACAC |
| 101 | CGGAATAACAATGAAATAGCAATAAAACAGGG |
| 102 | TGGCAACACCTTTTTAAGAAAAGTAAATAGCA |
| 103 | GTTAGCAAAAAGTTACCAGAAGGAAGAAACGA |
| 104 | AAGGGCGAAAAGACAA |
| 105 | CGATTGAGCATCTTTTCATAATCAAGCCACCA |
| 106 | AATTATTCTTTTCGGTCATAGCCCCCACCCTC |
| 107 | CCGACTTGTAGCGTCAGACTGTAGACCCTCAG |
| 108 | AAAATCACGTAATCAGTAGCGACAAGCCGCCGCCAGCATTGACAGG |
| 109 | GCGTTTGCGGAGGGAAGGTAAATATCAATAGA |
| 110 | CATCGGCAATTAAAGGTGAATTATAGACACCA |
| 111 | TTTGCCTTAGCCATTTGGGAATTACATAAAGG |
| 112 | ATAGCAGCACCCAGTAGCACCATTACCCGCAGTAT |
| 113 | ACGTCACCAATTTTTTTCCCTCAGAGCCAC |
| 114 | GGAACCAGAAATCACC |
| 115 | CCGGAACCGCCTCCCTCAGAGCCGCCTTATTA |
| 116 | AGAACCGCCACCCTCAGAGCCACCCGCGTTTT |
| 117 | AGCCGCCACCAGAACCACCACCAGGAATCAAG |
| 118 | CGTTCCAGTAAGCGTCGGATTAGCCCAGGCGG |
| 119 | GCCAGAATGGAAAGCGCAGTCTCTATAAGTAT |
| 120 | TGATATTCACAAACAAATAAATCCACTCAGGA |
| 121 | GTTGAGGCAGGTCAGACGCCGCCACC |
| 122 | GCTCAGTAGGGGTTTT |
| 123 | ATAAGTGCAGCATTCCACAGACAGAACGATCT |
| 124 | AGCCCGGAGTTTCGTCACCAGTACTTAGTAAA |
| 125 | GGTTTAGTGCCCAATAGGAACCCATAAACAAC |
| 126 | CTCAGAACCGCCATTTTGAAACCATCG |
| 127 | ACGCCTGTCGTCGAGAGGGTTGATGAATTTAC |
| 128 | AACACTGAATAGGTGTATCACCGTTCATTAAA |
| 129 | GATAGCAAACCGCCACCCTCAGAAATTGGCCT |
| 130 | CACCCTCAAATAGAAAGGAACAAC |
| 131 | GTTAGCGTCCCTCATA |
| 132 | AAAGTTTTCAACCATCGCCCACGCGTCGCTGA |
| 133 | AAAGTTTTCAACCATCGCCCACGCGTCGCTGA |
| 134 | TTTCAACACAGCTTGCTTTCGAGGACAGCATC |
| 135 | AATAATAATTTTTTTTTACTCCTTATTAATTAGCAAGGCCGGAA |
| 136 | AATGACAAGTCGTCTTTCCAGACGAAACTACA |
| 137 | TTAAACAGCTGTATGGGATTTTGCTGTACCGT |
| 138 | CGGTTTATGTTTCAGCGGAGTGAGTTTTCAGG |
| 139 | TCCAAAAGGAGCCTTTGAGGCTTTGAGGACTA |
| 140 | TCTCCAAAAAAAAGGCTAAAGGAATTGCGCACGTTGAAAAGAAGTTTCCATTTTAAAAGAACTGGCATGATAATAAC |
| 141 | GGAATACCC |
| 142 | TATATTCGATAACCGA |
| 143 | GGCTTGCAGCGACCTGCTCCATGTGGCGCAGA |
| 144 | GATCGTCAGTATCATCGCCTGATATGACCAAC |
| 145 | GGAACGAGACCAAGCGCGAAACAAGTACAGAC |
| 146 | TAAACCAACC |
| 147 | CGAAATCCGGGAGTTAAAGGCCGCGCGCCGAC |
| 148 | GGAGATTTCCCTCAGCAGCGAAAGTGAATTTC |
| 149 | GCGATTATGGTAGCAACGGCTACAAATTGTAT |
| 150 | TAAAACACTCATCTTTCATCAAGAGTAATCTT |
| 151 | AGGCAAAAGAATACACAAGACTTTTTCATGAGGCCACTACGAAGGCACGGGTAAAATACGTAATAAACAGCCATATTATT |
| 152 | TTGCCAGTTACAAAAT |
| 153 | CGGAACGATACTTAGC |
| 154 | CGGTCAATGCGATTTTAAGAACTGAGGACGTT |
| 155 | TTTGAAAGATTTCAACTTTAATCAAACGAACT |
| 156 | CAGGCGCAAACGAGTAGTAAATTGGAAAGATT |
| 157 | TACCTTATCATAAGGGAACCGAACAATTGTGT |
| 158 | ATGGTTTAAGGACAGATGAACGGTAGTACAAC |
| 159 | AACACCAGTAGGCTGGCTGACCTTGACCCCCA |
| 160 | TGAATAAGGCTTGCCCACATTCAA |
| 161 | GCTGCTCATTCAGGACAAGAACCGGATAT |
| 162 | TACCAGTCGCTCATTA |
| 163 | GGGAAGAAAAGAAGTTTTGCCAGATGTTTAGA |
| 164 | AACGGAACATAAAAACCAAAATAGATCGTCAT |
| 165 | CATCAGTTACACTATCATAACCCTATGCTTTA |
| 166 | CTAATGCAGATACTTTTTTAGTTTCATTCC |
| 167 | TTTTGCAAAAATCTACGTTAATAATTGTGAAT |
| 168 | AGACGACGAACATTATTACAGGTAGGCTTGAG |
| 169 | TAAGAGCAGAGATTTAGGAATACCTGACGAGA |
| 170 | GAATTACGCATAAATCAAAAATCAGGTCTT |
| 171 | TAGTAAAAGGGGGTAA |
| 172 | CTGGATAGCGTCCAATACTGCGGACGAGAGGC |
| 173 | AAATATTCATTGAATCCCCCTCAACGTTTACC |
| 174 | AACAGTTCAGAAAACGAGAATGACAGGCATAG |
| 175 | GCTTCAAAGCGAACCAGACCGGAACAGGATTA |
| 176 | CGAAAGACTTCAAATATCGCGTTTTGATAAGA |
| 177 | GCGGATTGCATCAAAAAGATTAAGGCTTAATT |
| 178 | CCCTGACTATTATAGTCACATGTTTT |
| 179 | CAACAGGTGCAAACTC |
| 180 | GAGAGTACATTCTACTAATAGTAGTAAATCAT |
| 181 | GGTCATTTCATTTGGGGCGCGAGCAATTAAGC |
| 182 | GCTGAATACAAATGGTCAATAACCAAATCGGT |
| 183 | AAATATGCGTAGATTTAGTTTGACTAATACTT |
| 184 | GGTGTCTGGATTTTATAACGCCAAAAG |
| 185 | TGGCATCACTTTAATTGCTCCTTTTAATTCGA |
| 186 | TATATTTTTTGCGGATGGCTTAGAAGGAAGCC |
| 187 | ACATTTCGTAATGCTGTAGCTCAAGAAGCAAA |
| 188 | GCGAACGAAACTAAAGTAC |
| 189 | ATATAACAGTTGATTCCGCAAGGATAATTTATCAACGTAACAAA |
| 190 | ACATCCAATAGCATTA |
| 191 | ACAGGCAATCTACAAAGGCTATCATCTGGAGC |
| 192 | AATAAAGCTAATGCCGGAGAGGGTATCGTAAA |
| 193 | TGTACCAAAATATGATATTCAACCCCGGTTGA |
| 194 | TTGCGGGATGAGAAAGGCCGGAGAGAAGATTG |
| 195 | AAATTCTGAGTAATGTGTAGG |
| 196 | TTGAGAGAGGCAAAGAATTAGCAATGAAAAGG |
| 197 | TGATAAATCTCAGAGCATAAAGCTTGTTTAGC |
| 198 | TCACCATCAAACATTATGACCCTGCATTAGAT |
| 199 | CAAAAGGGGAAGCCTTTATTTCAACCAATTCTATTTTAAATGCAATGCTTTAGAACCCTCATATTTTTAAAACGAAAGTCA |
| 200 | TTACCCAA |
| 201 | CCTGAGAGGGTCATTG |
| 202 | AAACAAGATGACCGTAATGGGATAATGGGCGC |
| 203 | ACTAGCATCGTCGGATTCTCCGTGTGAGGGGA |
| 204 | TAATCAGATCATCAACATTAAATGGATCGCAC |
| 205 | TATAAGCAAATATTTAAATTGTAATAAAGATT |
| 206 | ACGTTAATATTTTGTTCATTTTTTAACCAATA |
| 207 | CGGCGGATGAATCGATGAACGGTAAGCTATTT |
| 208 | TAACAACCGTCAATCATATGTACCGTTCTAGC |
| 209 | GCCAGCTTAAAGCCCCAAAAACAGCAGTCAAA |
| 210 | ATCAAAAATAATTCGCGTCTGGCCTCTGGTGC |
| 211 | TGGTGTAGGGTCACGT |
| 212 | ATCGTAACACTCTAGAGGATCCCCTTCGTAAT |
| 213 | CGACGACAGGCCAGTGCCAAGCTTGAAATTGT |
| 214 | TCCAGCCAGGGTTTTCCCAGTCACATACGAGC |
| 215 | CGGAAACCGTGCTGCAAGGCGATTGGGTGCCT |
| 216 | GCCATTCAGGCTGCGCTTCGC |
| 217 | GCAGGTCGCGTGCATCTGCCAGTTGGAACAAA |
| 218 | AAAACGACGTATCGGCCTCAGGAATGAGCGAG |
| 219 | TAACGCCAGCTTTCCGGCACCGCTTTCCTGTA |
| 220 | AGGGGGATAGGCAAAGCGCCATTCGGAACGCC |
| 221 | AGCTCGAAGGGTACCG |
| 222 | CATGGTCATGATTGCCCTTCACCGTTGCAGCA |
| 223 | TATCCGCTTTTTTCTTTTCACCAGGCAGGCGA |
| 224 | CGGAAGCAAGAGGCGGTTTGCGTAAAATCGGC |
| 225 | AATGAGTGAGCTGCATTAATGAATTAGCCCGAGATAGGGTTGAGTG |
| 226 | GCAACAGCTAGCTGTTTCCTGTGTGCATGCCT |
| 227 | CAGGGTGGCACAATTCCACACAACGACGTTGT |
| 228 | GCGCGGGGTAAAGTGTAAAGCCTGAAGTTGGG |
| 229 | CCTGTCGTGCCAGCTAACTCACATTAACTGGCGAA |
| 230 | GCCCGCTTTCCTTTTTTCGCTGGCAAGTGT |
| 231 | TGAGAGAGCCTGGCCC |
| 232 | AGCGGTCCACGCTGGTTTGCCCCATGAGACGG |
| 233 | AAATCCTGTTTGATGGTGGTTCCGTTGGGCGC |
| 234 | AAAATCCCTTATAAATCAAAAGAACGGCCAAC |

**Table S11.****Serological examination of patients bitten by ticks for MKWV infection.**

| Province | Patient No. | Eilsa | | |  | VNT |
| --- | --- | --- | --- | --- | --- | --- |
|  |  | IgG |  | IgM |  |  |
| Inner Mongolia | 1 | 0.224 |  | 0.189 |  | — |
|  | 2 | 0.31 |  | 0.241 |  | — |
|  | 3 | 0.216 |  | 0.209 |  | — |
|  | 4 | 0.395 |  | 0.169 |  | — |
|  | 5 | 0.25 |  | 0.211 |  | — |
|  | 6 | 0.274 |  | 0.172 |  | — |
|  | 7 | 0.25 |  | 0.217 |  | — |
|  | 8 | 0.49 |  | 0.197 |  | — |
|  | 9 | 0.363 |  | 0.184 |  | — |
|  | 10 | 0.235 |  | 0.164 |  | — |
|  | 11 | 0.216 |  | 0.176 |  | — |
|  | 12 | 0.221 |  | 0.122 |  | — |
|  | 13 | 0.354 |  | 0.093 |  | — |
|  | 14 | 0.287 |  | 0.142 |  | — |
|  | 15 | 0.25 |  | 0.191 |  | — |
|  | 16 | 0.276 |  | 0.187 |  | — |
|  | 17 | 0.342 |  | 0.09 |  | — |
|  | 18 | 0.323 |  | 0.135 |  | — |
|  | 19 | 0.221 |  | 0.224 |  | — |
|  | 20 | 0.233 |  | 0.243 |  | — |
|  | 21 | 0.305 |  | 0.19 |  | — |
|  | 22 | 0.288 |  | 0.175 |  | — |
|  | 23 | 0.38 |  | 0.184 |  | — |
|  | 24 | 0.247 |  | 0.17 |  | — |
|  | 25 | 0.42 |  | 0.169 |  | — |
|  | 26 | 0.329 |  | 0.155 |  | — |
|  | 27 | 0.325 |  | 0.175 |  | — |
|  | 28 | 0.265 |  | 0.16 |  | — |
|  | 29 | 0.271 |  | 0.189 |  | — |
|  | 30 | 0.298 |  | 0.186 |  | — |
|  | 31 | 0.366 |  | 0.158 |  | — |
|  | 32 | 0.378 |  | 0.14 |  | — |
|  | 33 | 0.331 |  | 0.129 |  | — |
|  | 34 | 0.231 |  | 0.138 |  | — |
|  | 35 | 0.328 |  | 0.192 |  | — |
|  | 36 | 0.252 |  | 0.18 |  | — |
|  | 37 | 0.402 |  | 0.204 |  | — |
|  | 38 | 0.311 |  | 0.161 |  | — |
|  | 39 | 0.214 |  | 0.134 |  | — |
|  | 40 | 0.242 |  | 0.168 |  | — |
|  | 41 | 0.263 |  | 0.219 |  | — |
|  | 42 | 0.31 |  | 0.178 |  | — |
|  | 43 | 0.207 |  | 0.163 |  | — |
|  | 44 | 0.551 |  | 0.211 |  | — |
|  | 45 | 0.183 |  | 0.137 |  | — |
|  | 46 | 0.294 |  | 0.173 |  | — |
|  | 47 | 0.255 |  | 0.134 |  | — |
|  | 48 | 0.437 |  | 0.128 |  | — |
|  | 49 | 0.341 |  | 0.139 |  | — |
|  | 50 | 0.291 |  | 0.139 |  | — |
|  | 51 | 0.422 |  | 0.119 |  | — |
|  | 52 | 0.167 |  | 0.144 |  | — |
|  | 53 | 0.257 |  | 0.171 |  | — |
|  | 54 | 0.212 |  | 0.146 |  | — |
|  | 55 | 0.266 |  | 0.186 |  | — |
|  | 56 | 0.306 |  | 0.195 |  | — |
|  | 57 | 0.327 |  | 0.105 |  | — |
|  | 58 | 0.335 |  | 0.165 |  | — |
|  | 59 | 0.31 |  | 0.169 |  | — |
|  | 60 | 0.305 |  | 0.176 |  | — |
|  | 61 | 0.462 |  | 0.144 |  | — |
|  | 62 | 0.278 |  | 0.13 |  | — |
|  | 63 | 0.373 |  | 0.111 |  | — |
|  | 64 | 0.297 |  | 0.096 |  | — |
|  | 65 | 0.334 |  | 0.164 |  | — |
|  | 66 | 0.368 |  | 0.132 |  | — |
|  | 67 | 0.304 |  | 0.146 |  | — |
|  | 68 | 0.208 |  | 0.138 |  | — |
|  | 69 | 0.234 |  | 0.177 |  | — |
|  | 70 | 0.247 |  | 0.189 |  | — |
|  | 71 | 0.234 |  | 0.128 |  | — |
|  | 72 | 0.195 |  | 0.206 |  | — |
|  | 73 | 0.222 |  | 0.175 |  | — |
|  | 74 | 0.426 |  | 0.151 |  | — |
|  | 75 | 0.407 |  | 0.174 |  | — |
|  | 76 | 0.323 |  | 0.15 |  | — |
|  | 77 | 0.26 |  | 0.17 |  | — |
|  | 78 | 0.25 |  | 0.179 |  | — |
|  | 79 | 0.226 |  | 0.135 |  | — |
|  | 80 | 0.334 |  | 0.121 |  | — |
|  | 81 | 0.197 |  | 0.186 |  | — |
|  | 82 | 0.194 |  | 0.158 |  | — |
|  | 83 | 0.256 |  | 0.114 |  | — |
|  | 84 | 0.258 |  | 0.167 |  | — |
|  | 85 | 0.26 |  | 0.142 |  | — |
|  | 86 | 0.239 |  | 0.15 |  | — |
|  | 87 | 0.328 |  | 0.199 |  | — |
|  | 88 | 0.244 |  | 0.177 |  | — |
|  | 89 | 0.249 |  | 0.131 |  | — |
|  | 90 | 0.223 |  | 0.189 |  | — |
|  | 91 | 0.283 |  | 0.164 |  | — |
|  | 92 | 0.383 |  | 0.152 |  | — |
|  | 93 | 0.299 |  | 0.202 |  | — |
|  | 94 | 0.274 |  | 0.147 |  | — |
|  | 95 | 0.276 |  | 0.147 |  | — |
|  | 96 | 0.372 |  | 0.211 |  | — |
|  | 97 | 0.461 |  | 0.142 |  | — |
|  | 98 | 0.41 |  | 0.148 |  | — |
|  | 99 | 0.476 |  | 0.126 |  | — |
|  | 101 | 0.393 |  | 0.153 |  | — |
|  | 102 | 0.307 |  | 0.144 |  | — |
|  | 103 | 0.333 |  | 0.147 |  | — |
|  | 104 | 0.398 |  | 0.144 |  | — |
|  | 105 | 0.394 |  | 0.152 |  | — |
|  | 106 | 0.353 |  | 0.159 |  | — |
|  | 107 | 0.335 |  | 0.199 |  | — |
|  | 108 | 0.531 |  | 0.23 |  | 1:40 |
|  | 109 | 0.59 |  | 0.19 |  | 1:80 |
|  | 110 | 0.39 |  | 0.148 |  | — |
|  | 111 | 0.25 |  | 0.162 |  | — |
|  | 112 | 0.403 |  | 0.186 |  | — |
|  | 113 | 0.212 |  | 0.088 |  | — |
|  | 114 | 0.339 |  | 0.099 |  | — |
|  | 115 | 0.279 |  | 0.146 |  | — |
| Jilin | 1 | 0.404 |  | 0.094 |  | — |
|  | 2 | 0.518 |  | 0.148 |  | 1:40 |
|  | 3 | 0.44 |  | 0.168 |  | — |
|  | 4 | 0.623 |  | 0.177 |  | 1:160 |
|  | 5 | 0.462 |  | 0.133 |  | — |
|  | 6 | 0.483 |  | 0.11 |  | — |
|  | 7 | 0.384 |  | 0.151 |  | — |
|  | 8 | 0.501 |  | 0.118 |  | — |
|  | 9 | 0.622 |  | 0.183 |  | 1:20 |
|  | 10 | 0.569 |  | 0.097 |  | — |
|  | 11 | 0.345 |  | 0.109 |  | — |
|  | 12 | 0.52 |  | 0.137 |  | — |
|  | 13 | 0.406 |  | 0.151 |  | — |
|  | 14 | 0.364 |  | 0.132 |  | — |
|  | 15 | 0.368 |  | 0.201 |  | — |
|  | 16 | 0.451 |  | 0.188 |  | — |
|  | 17 | 0.363 |  | 0.084 |  | — |
|  | 18 | 0.307 |  | 0.187 |  | — |
|  | 19 | 0.352 |  | 0.101 |  | — |
|  | 20 | 0.369 |  | 0.111 |  | — |
|  | 21 | 0.604 |  | 0.142 |  | — |
|  | 22 | 0.094 |  | 0.147 |  | — |
|  | 23 | 0.237 |  | 0.126 |  | — |
|  | 24 | 0.388 |  | 0.142 |  | — |
|  | 25 | 0.404 |  | 0.087 |  | — |
|  | 26 | 0.283 |  | 0.101 |  | — |
|  | 27 | 0.391 |  | 0.09 |  | — |
|  | 28 | 0.371 |  | 0.077 |  | — |
|  | 29 | 0.245 |  | 0.152 |  | — |
|  | 30 | 0.418 |  | 0.113 |  | — |
| Negative control | 1 | 0.403 |  | 0.177 |  | — |
|  | 2 | 0.261 |  | 0.15 |  | — |
|  | 3 | 0.207 |  | 0.121 |  | — |
|  | 4 | 0.289 |  | 0.14 |  | — |
|  | 5 | 0.251 |  | 0.118 |  | — |
|  | 6 | 0.319 |  | 0.069 |  | — |
|  | 7 | 0.225 |  | 0.076 |  | — |
|  | 8 | 0.214 |  | 0.063 |  | — |
|  | 9 | 0.127 |  | 0.063 |  | — |
|  | 10 | 0.188 |  | 0.072 |  | — |
